# Novel Assay Platform to Evaluate Intracellular Killing of *Mycobacterium tuberculosis*: *In Vitro* and *In Vivo* Validation

**DOI:** 10.3389/fimmu.2021.750496

**Published:** 2021-11-12

**Authors:** Kata Horváti, Kinga Fodor, Bernadett Pályi, Judit Henczkó, Gyula Balka, Gergő Gyulai, Éva Kiss, Beáta Biri-Kovács, Zsuzsanna Senoner, Szilvia Bősze

**Affiliations:** ^1^ Eötvös Loránd Kutatási Hálózat-Eötvös Loránd Tudományegyetem (ELKH-ELTE) Research Group of Peptide Chemistry, Eötvös Loránd Research Network, Eötvös Loránd University, Budapest, Hungary; ^2^ Institute of Chemistry, Eötvös Loránd University, Budapest, Hungary; ^3^ Department of Laboratory Animal Science and Animal Protection, University of Veterinary Medicine, Budapest, Hungary; ^4^ National Biosafety Laboratory, National Public Health Center, Budapest, Hungary; ^5^ Department of Pathology, University of Veterinary Medicine, Budapest, Hungary; ^6^ Laboratory of Interfaces and Nanostructures, Institute of Chemistry, Eötvös Loránd University, Budapest, Hungary; ^7^ National Korányi Institute of Pulmonology, Budapest, Hungary

**Keywords:** tuberculosis, cell penetrating peptides, isoniazid, Dhvar4, Transwell, spheroid, MonoMac-6

## Abstract

One of the main hallmarks of tuberculosis (TB) is the ability of the causative agent to transform into a stage of dormancy and the capability of long persistence in the host phagocytes. It is believed that approximately one-third of the population of the world is latently infected with *Mycobacterium tuberculosis* (*Mtb*), and 5%–10% of these individuals can develop clinical manifestations of active TB even decades after the initial infection. In this latent, intracellular form, the *bacillus* is shielded by an extremely robust cell wall and becomes phenotypically resistant to most antituberculars. Therefore, there is a clear rationale to develop novel compounds or carrier-conjugated constructs of existing drugs that are effective against the intracellular form of the bacilli. In this paper, we describe an experimental road map to define optimal candidates against intracellular *Mtb* and potential compounds effective in the therapy of latent TB. To validate our approach, isoniazid, a first-line antitubercular drug was employed, which is active against extracellular *Mtb* in the submicromolar range, but ineffective against the intracellular form of the bacteria. Cationic peptide conjugates of isoniazid were synthesized and employed to study the host-directed drug delivery. To measure the intracellular killing activity of the compounds, *Mtb*-infected MonoMac-6 human monocytic cells were utilized. We have assessed the antitubercular activity, cytotoxicity, membrane interactions in combination with internalization efficacy, localization, and penetration ability on interface and tissue-mimicking 3D models. Based on these *in vitro* data, most active compounds were further evaluated *in vivo* in a murine model of TB. Intraperitoneal infectious route was employed to induce a course of slowly progressive and systemic disease. The well-being of the animals, monitored by the body weight, allows a prolonged experimental setup and provides a great opportunity to test the long-term activity of the drug candidates. Having shown the great potency of this simple and suitable experimental design for antimicrobial research, the proposed novel assay platform could be used in the future to develop further innovative and highly effective antituberculars.

## Introduction

Tuberculosis (TB) is the leading infectious disease caused by a single pathogen, responsible for 1.4 million deaths annually (1, 2). It is estimated that more than one-third of the population of the world is latently infected with the causative agent *Mycobacterium tuberculosis* (*Mtb*), leading to a state of latent TB infection (LTBI) (2). LTBI is defined as the presence of *Mtb*-specific immune response in the absence of TB symptoms. A full spectrum of TB disease states can be drawn between the asymptomatic and symptomatic clinical manifestation, including bacterial clearance, persistence, and reactivation (3–5). The reduced efficacy of the currently applied chemotherapy concurrent with the high prevalence of multidrug-resistant TB calls for new drug compounds with novel mode of action.

Protocols utilized for TB research differ in the route of infection, inoculum, dose, and strain of the bacteria, route of drug administration, timing, and type of endpoint analysis. Different preclinical animal models have been employed, of which mouse is the most preferred experimental model for many practical reasons. The small size and cost-effectiveness, together with the availability of more abundant commercial reagents, immunological evaluation indices and genetically modified strains, are among the reasons why more than 60% of the studies utilize mice for *in vivo* TB experiments (6). However, guinea pig is generally considered a more accurate model because the lung pathology is closer to human TB, including the granuloma formation (7, 8). Nonhuman primates represent an excellent model of human TB because of their susceptibility to *Mtb* infection, and they can develop a full spectrum of the disease (9). However, nonhuman primates can be only employed at a later phase of research. Rabbits were successfully utilized as a model of LTBI and to study bacillary control and reactivation of the disease (10, 11). However, the limited availability of immunological reagents represents a challenge. Zebrafish (*Danio rerio*) has become an attractive vertebrate model for studying mycobacterial infection and screening antibacterial drug candidates. However, the need for special facilities represents a difficulty for most of the laboratories (12–14).

Recent mice studies have shown that intraperitoneal or intradermal injection of *Mtb* leads to a low-grade infection showing containment mainly in the draining lymph node and the spleen (15, 16). Low-dose *i.p.* or *i.d*. infection approaches are used to model LTBI in humans to study novel intervention strategies and intracellular antitubercular efficacy of drug compounds.

In humans, the infection by inhaling small aerosol droplets containing *Mtb*, is followed by the phagocytosis of alveolar macrophages and dendritic cells. By analyzing the sputum of TB patients, neutrophils were identified as the predominant *Mtb*-infected phagocytic cells (17). *Mtb* enters the phagocytes by receptor-mediated endocytosis through various receptors such as Fc-, mannose-, and C-type lectin receptors (18–21). Some receptors allow the so-called silent entry, while others induce defense mechanisms (22). After internalization, *Mtb*-infected phagosomes should interact with early than late endocytic organelles and undergo phagosomal maturation. However, *Mtb* can escape immunity by utilizing several distinct mechanisms of action such as the inhibition of the formation of lysosomes, acidification, phagosomal maturation, cytokine-mediated macrophage activation, and apoptosis (23).

**Figure 1:**
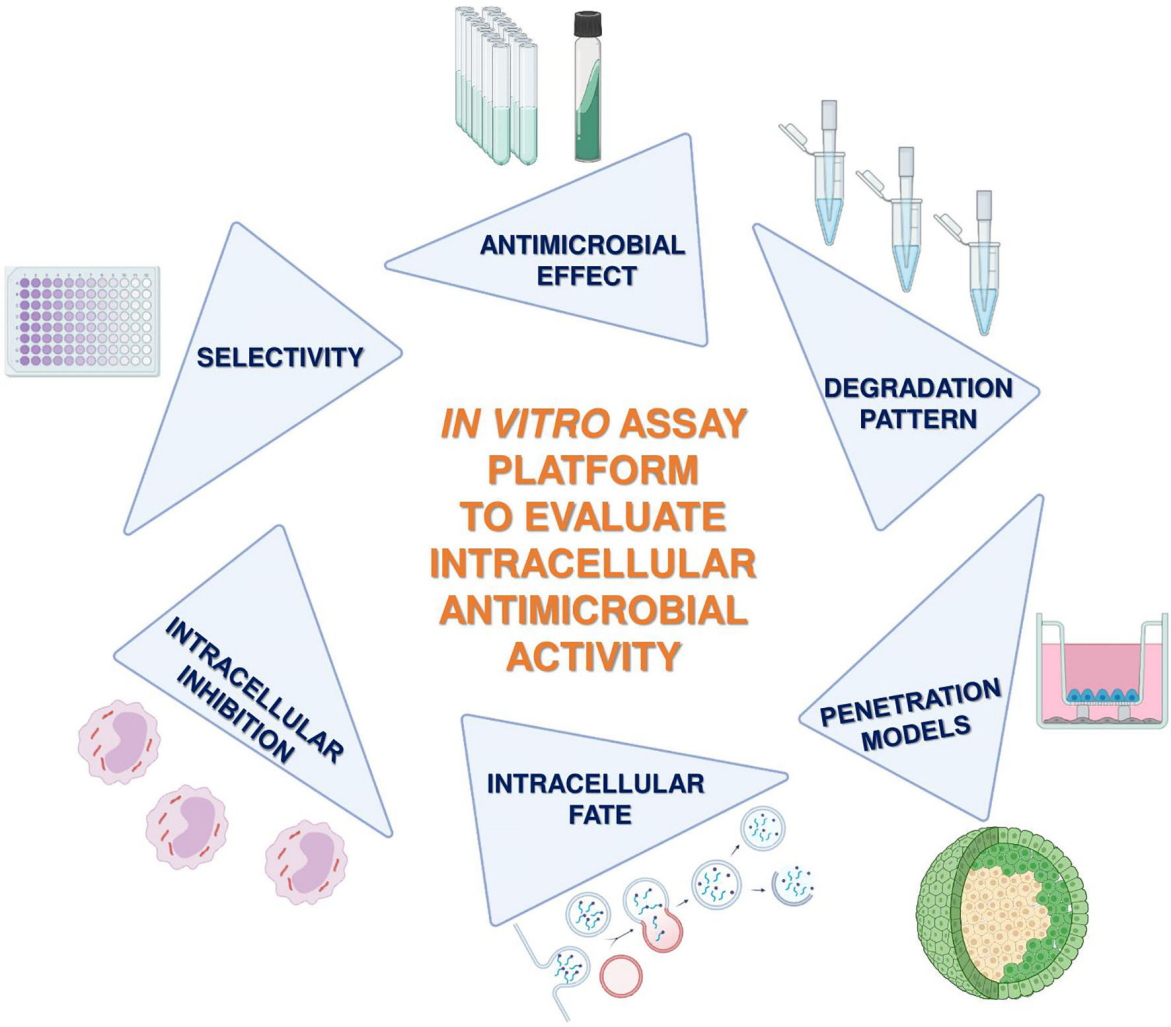
Schematic flow chart of the proposed assay platform to define optimal candidates against intracellular pathogens. The main steps of the experimental setup: (i) activity on extracellular bacteria, (ii) determination of selectivity on potential human host cells, (iii) intracellular killing efficacy, (iv) internalization and intracellular localization, (v) penetration ability on tissue-mimicking 3D spheroids and on Transwell-based noncontact coculture interfaces, and (vi) degradation patterns.

The great survival ability of *Mtb* and its success to cause systemic latent disease is based on the ability to transform into a stage of dormancy in which the bacterium changes its gene expression, builds an extremely robust cell wall, and develops resistance to most of the used drug compounds (22). Successful adaptation of *Mtb* to the intracellular environment of the phagocytes is the leading cause of failure for drugs against LTBI and TB. Traditional extracellular bacterial culture systems, that were used to identify antitubercular candidates, are limited by their low level of complexity, and they also cannot reflect on the intracellular status of *Mtb*. In this paper, a road map of experimental approaches to identify new compounds effective against TB and LTBI is proposed **(** **Figure 1**). An optimal candidate (i) must be effective against *Mtb* and (ii) must reach its target inside the *Mtb*-infected phagocytes in its active form and (iii) its compound should not be toxic to the host cells, (iv) must avoid endosomal entrapment, (v) must not efflux out, and (vi) must be permeable to the concerned tissue. Human cellular systems, together with state-of-the-art human-based tissue model systems, allow us to open new avenues to investigate better-acting antituberculars.

Intracellular assays that quantify the bacterial survival inside the host phagocytes are used as host-based screening platforms to describe new, better-acting drugs (24). These screens were performed with *Mtb* or its surrogate *M. bovis* BCG (25) residing mainly in macrophages. Fluorescent reporter *Mtb* strains have additional benefits in visualizing the host-*Mtb* interaction and infection mechanisms (26). Also, inducible reporters are extensively studied, which fluoresce on exposure to a particular environmental change such as low pH or hypoxia (27). Recently, multicolor reporter strains were successfully utilized to simultaneously detect changes in the protein expression and intracellular ion concentrations (28). As host cell lines, murine J774A.1 (29, 30), murine RAW 264.7 (31, 32), and human THP-1 (33, 34) were frequently used in recently published assays. In our laboratory, MonoMac-6 human monocytes were studied to broaden the spectrum of host systems for *Mtb* (35, 36). The ease of maintenance and the complete capture of the target pathogen made MonoMac-6 a perfect choice for compound screening against intracellular *Mtb*.

Compared with traditional methods employing two-dimensional monolayer cultures, three-dimensional (3D) cell culture systems are recognized as they more accurately model physiological cellular functions and cell-to-cell interactions. Due to mimicking the assembly of the tissue or organ environment, 3D systems show advantages in providing more predictive data for *in vivo* studies (37). The 3D spheroids are suitable and simple objects to characterize the penetration ability of the delivery peptides and drug conjugates.

Physical barriers limit the efficacy of drugs and delivery devices. To comparatively estimate penetration ability *via* tissue barriers, Transwell arrangements are promising models (38). As a bronchial interface model, we have studied the noncontact, submerged coculture Transwell system to compare the trafficking of the compounds through cellular barriers.

As a model compound to accomplish this experimental road map, isoniazid (INH) was applied because INH is active against *Mtb* in the submicromolar range but ineffective against the intracellular form of the bacteria even at 1,000 times higher treatment concentration (39–41). Targeting INH to the site of action inside the *Mtb* infected phagocytes and tissues holds promise to improve the drug efficacy and reduce the side effects.

Cell-penetrating peptides (CPPs) and antimicrobial peptides (AMPs) have a recent history in designing and developing of host-directed therapy against intracellular pathogens. CPPs and AMPs can cross the lipid membrane, access intracellular targets, and serve as carriers for small molecular drug compounds (42, 43). In the resistance era, it is important to note that AMPs have received extensive attention because they are typically not causing widespread resistance due to their simultaneous attack on the cell wall and intracellular targets. In this study, a representative set of CPPs and AMPs were applied as carrier peptides for INH.

## Material and Methods

### Materials

Amino acid derivatives and resins were obtained from Iris Biotech (Marktredwitz, Germany). Reagents, such as *N,N′*-diisopropylcarbodiimide (DIC), triisopropylsilane (TIS), 1-hydroxybenzotriazole (HOBt), 1,8-diazabicyclo[5.4.0]undec-7-ene (DBU), Isoniazid (INH), glyoxylic acid and 5(6)-carboxyfluorescein (*Cf*) were purchased from Sigma (Budapest, Hungary). Trifluoroacetic acid (TFA) and acetonitrile (AcN) were from VWR (Budapest, Hungary). *N,N*-dimethylformamide (DMF), dichlormethane (DCM), diethyl ether, and ethanol were purchased from Reanal (Budapest, Hungary)

For the *in vitro* assays, RPMI-1640 medium, fetal calf serum (FCS), and trypan blue were obtained from Sigma (Budapest, Hungary), while DMEM medium and 2 mM l-glutamine were from Lonza (Basel, Switzerland). Trypsin, nonessential amino acids, and penicillin-streptomycin were from Gibco (Thermo Fisher Scientific, Waltham, MA, USA). HPMI buffer (9 mM glucose, 10 mM NaHCO_3_, 119 mM NaCl, 9 mM HEPES, 5 mM KCl, 0.85 mM MgCl_2_, 0.053 mM CaCl_2_, 5 mM Na_2_HPO_4_ × 2H_2_O, pH = 7.4) was prepared in-house using components obtained from Sigma (Budapest, Hungary). BBL Löwenstein-Jensen medium was from Becton Dickinson (Környe, Hungary).

### Peptide Synthesis and Purification

Peptides were produced on solid phase (Fmoc-Rink Amide MBHA, capacity = 0.67 mmol/g) resin in an automated peptide synthesizer (Syro-I, Biotage, Uppsala, Sweden) using standard Fmoc/tBu strategy with DIC/HOBt coupling reagents. *Cf* was coupled to the *N*-terminus of the peptides by using DIC/HOBt coupling method. Peptides were cleaved from the resin with TFA/H_2_O/TIS (9.5:2.5:2.5, *v*/*v*) mixture (2 h, RT). After filtration, compounds were precipitated in cold diethyl ether, centrifuged (4,000 rpm, 5 min) and freeze-dried from water.

RP-HPLC purification was performed on an UltiMate 3000 Semiprep HPLC (Thermo Fisher Scientific) with a Phenomenex Jupiter Proteo C-12 column (250 × 10 mm) using gradient elution, consisting of 0.1% TFA in water (eluent A) and 0.1% TFA in acetonitrile/water = 80/20 (*v*/*v*) (eluent B).

Purified peptides were analyzed by LC-MS using a Thermo Scientific Q Exactive Focus Hybrid Quadrupole-Orbitrap Mass Spectrometer. For the separation, a Waters Acquity UPLC BEH C18 (1.7 µm, 150 × 2.1 mm) column was used with a flow rate of 0.3 ml/min.

Residual TFA and TFA counter-ion was removed by using an acetate-exchange resin. For the detailed description, see **Supplementary Information 1.1**).

### Conjugation of Isoniazid

Prior to conjugation, INH was derivatized as described previously (44). Briefly, 20.0 g, 0.146 mol INH (M = 137.1) was dissolved in 200 ml acetonitrile/water (1:1, *v*/*v*)) and reacted with 13.6 g, 0.148 mol glyoxylic acid monohydrate (M = 92.1, 1.01 equiv, dissolved in 50 ml water). After 1 h of stirring, the precipitate was filtered, washed with water and acetonitrile, and dried over P_2_O_5_ under vacuum (27.1 g, 96% yield, ESI MS calcd. 193.1 (Mmo) found 193.1).

The resulted isonicotinoylhydrazonoacetic acid (3.415 g, 17.7 mmol, M = 193.2) was reduced with 1.110 g, 17.7 mmol NaBH_3_CN (M = 62.8) in 30 ml abs. ethanol (12 h stirring, RT). The obtained yellowish solution was filtered, evaporated to dryness, and recrystallized from methanol (2.777 g, 81.2% yield, ESI MS calcd. 195.1 (Mmo) found 195.1)

The product, isonicotinoylhydrazinoacetic acid, (7 equiv) was added to the peptidyl resins described above, in the presence of 7 equiv. of DIC and HOBt (2 h, RT). INH-peptide conjugates were then cleaved from the resin with TFA in the presence of scavengers (2.5% H_2_O, 2.5% TIS; 2 h, RT), precipitated with cold diethyl ether, dissolved in water, freeze-dried, and purified as mentioned above.

### Cells, Bacteria, and Culture Conditions

MonoMac-6 human monocytic cell line (45) (DSMZ No. ACC 124, Braunschweig, Germany) was maintained as an adherent culture in RPMI-1640 supplemented with 10% heat-inactivated fetal calf serum (FCS) l-glutamine (2 mM) and gentamicin (35 μM) at 37°C in a humidified atmosphere containing 5% CO_2_.

EBC-1 lung squamous carcinoma (RRID: CVCL_2891) (46–48) and CALU-1 human lung epidermoid carcinoma (Sigma 93120818) (49, 50) were maintained in DMEM medium containing 10% FBS and supplemented with 2 mM l-glutamine, 1% nonessential amino acids, 1 mM sodium pyruvate, and 1% penicillin-streptomycin (from 10,000 units penicillin and 10 mg streptomycin/ml). Cells were cultured at 37°C, 5% CO_2_-humidified atmosphere.

*Mycobacterium tuberculosis* H37Rv (ATCC 27294) was grown in Sauton’s liquid medium to exponential growth phase (approx. 3–4 weeks). The Sauton’s medium was prepared in-house as described in (51) with the addition of 0.05% (*w*/*v*) Tween-80 to prevent bacterial aggregation (since *Mtb* H37Rv tends to form clumps). The bacterial suspension was homogenized by ball-milling using sterilized stainless steel grinding balls, and after dilution, it was used for the inoculation of the test tubes.

All experimental procedures with infectious *Mtb* were performed in a biosafety level 3 (BSL-3) laboratory at the National Public Health Center (Hungary), respecting the institutional containment level 3 laboratory management and biosecurity standards based on applicable national and EU Directives.

### Determination of *In Vitro* Antitubercular Effect

Antimycobacterial activity of INH and INH-conjugates was determined on *Mtb* H37Rv using broth dilution method in Sula semisynthetic medium (pH = 6.5) (52–54). Dilution series of the compounds were prepared in DMSO and added to 5 ml Sula medium-containing test tubes. The *Mtb* bacterial suspension (0.5 McFarland equal to 1.5 × 10^8^ CFU/ml) were diluted 10^4^ times and added to the test tubes. The minimal inhibitory concentration (MIC, reported in micromolars) was determined after incubation at 37°C for 28 days. MIC was the lowest concentration of a compound at which no visible growth of the bacteria occurred. The antitubercular effect of the tested compounds was confirmed using a colony-forming unit (CFU) determination by subculturing 100 µl of the supernatant onto drug-free Löwenstein-Jensen solid medium (37°C, 28 days), which is a selective medium specifically used for the culture and isolation of *Mtb*. Experiments were repeated at least two times.

### Cellular Uptake and Intracellular Localization

The internalization of the compounds was measured in MonoMac-6 cells. For the assay, cells were treated with *Cf*-labeled peptides at 5, 10, and 20 µM final concentration and were incubated for 2 h. After centrifugation (1,000 rpm, 5 min) and washing with serum-free RPMI medium, the supernatant was removed, and 100 μl 0.25% trypsin was added to the cells. After 5 min incubation, 0.8 ml 10% FCS/HPMI medium was added, then cells were washed and resuspended in 0.3 ml HPMI medium. The intracellular fluorescence intensity of the cells was measured on a BD LSR II flow cytometer (BD Biosciences, Franklin Lakes, NJ, USA) on channel FITC (emission at *λ* = 505 nm), and data were analyzed with FACSDiva 5.0 software (BD Biosciences). All measurements were performed in triplicates, and the mean fluorescent intensity together with the standard error of the mean (SEM) was graphically presented. For analysis of statistical significance, unpaired *t*-test was used.

Cellular uptake and localization were visualized by confocal laser scanning microscopy. MonoMac-6 cells were seeded 1 day prior to treatment into coverslips (thickness 1, Assistent, Karl Hecht GmbH, Sondheim vor der Rhön, Germany) containing a 24-well plate (Sarstedt, Nümbrecht, Germany) for microscopy studies at a density of 7.5 × 10^4^ cells/well, in 1 ml complete DMEM medium. The following day, cells were treated with *Cf*-peptide at a concentration of 10 µM (diluted in serum-free DMEM medium) for 2 h. Lysosomes were stained by LysoTracker Deep Red (Invitrogen, Waltham, MA, USA) for 30 min, and nuclei were stained by Hoechst 33342 solution (Thermo Scientific, USA). After each step, cells were washed three times with serum-free medium, then cells were fixed by 4% paraformaldehyde for 15 min and mounted to microscopy slides with Mowiol 4-88 (Sigma, Budapest, Hungary). Imaging was performed by a Zeiss LSM-710 system (Carl Zeiss microscopy GmbH, Oberkochen, Germany) with a ×40/1.4 Plan-Apochromat oil immersion objective using lasers with the parameters: *Cf* peptides *λ*ex = 488 nm, *λ*em = 541 nm, nuclei *λ*ex = 405 nm, *λ*em = 467 nm (Hoechst 33342), lysosomes *λ*ex = 633 nm, *λ*em = 720 nm (LysoTrackerTM Deep Red) or *λ*ex = 577 nm, *λ*em = 590 nm (LysoTrackerTM Red DND-99) were used. Zeiss ZEN lite software (Carl Zeiss Microscopy GmbH, Jena, Germany) was used for image processing.

### Transwell Experiments to Determine Penetration Ability Through Bronchial Interface

EBC-1 lung squamous carcinoma and CALU-1 human lung epidermoid carcinoma were seeded on 24-well plates with Transwell inserts (TW) [Nunc, Sigma polycarbonate microporous membrane, 0.4 µm pore size (growth area 0.412 cm^2^)]. Before use, TW inserts were incubated with ICM. On day 1, 300 μl of CALU-1 suspension in complete DMEM medium (8.0 × 10^5^ cells) was pipetted onto the surface of the TW apical chamber and 500 μl complete DMEM medium was added to the basolateral side. In order to avoid nonattached cells, after incubation at 37°C for 5–6 h, the apical medium was removed and replaced with fresh complete DMEM medium. On day 3, the medium was changed, and CALU-1 cells were grown up to confluence (which was checked prior and after the experiments) with CellTracker Green (CMFDA (5-chloromethylfluorescein diacetate, Invitrogen, C2925). CMFDA was dissolved in serum-free DMEM medium to reach 10 µM concentration. After 35 min of incubation, the cells were washed with serum-free DMEM and cell nuclei of CALU-1 monolayers were stained with Hoechst 33342 solution, according to the manufacturer’s suggestions. ZEISS Axio Observer Inverse Imaging Platform (Zeiss) (equipped with Led3 470 blue, Led4 555 green; filter set: 25HE) was used to capture the TW insert membrane with CALU-1 monolayer. On day 5, the medium was changed, then *Cf*-peptide was added to the CALU-1-containing apical side at 10 and 20 µM concentrations, and the system was incubated for 45 min or 3 h (37°C, 5% CO_2_). The TW chamber was removed after incubation. EBC-1 cells were studied using flow cytometry (BD LSR II).

EBC-1 cells were treated without the presence of the TW inserts as controls (on day 4 prior to treatment, EBC-1 cells (10^5^ cells/well in complete DMEM).

### EBC-1 Spheroids as Lung Tissue-Mimicking Platform to Capture Penetration Profile of the *Cf* Peptides

**Figure 2:**
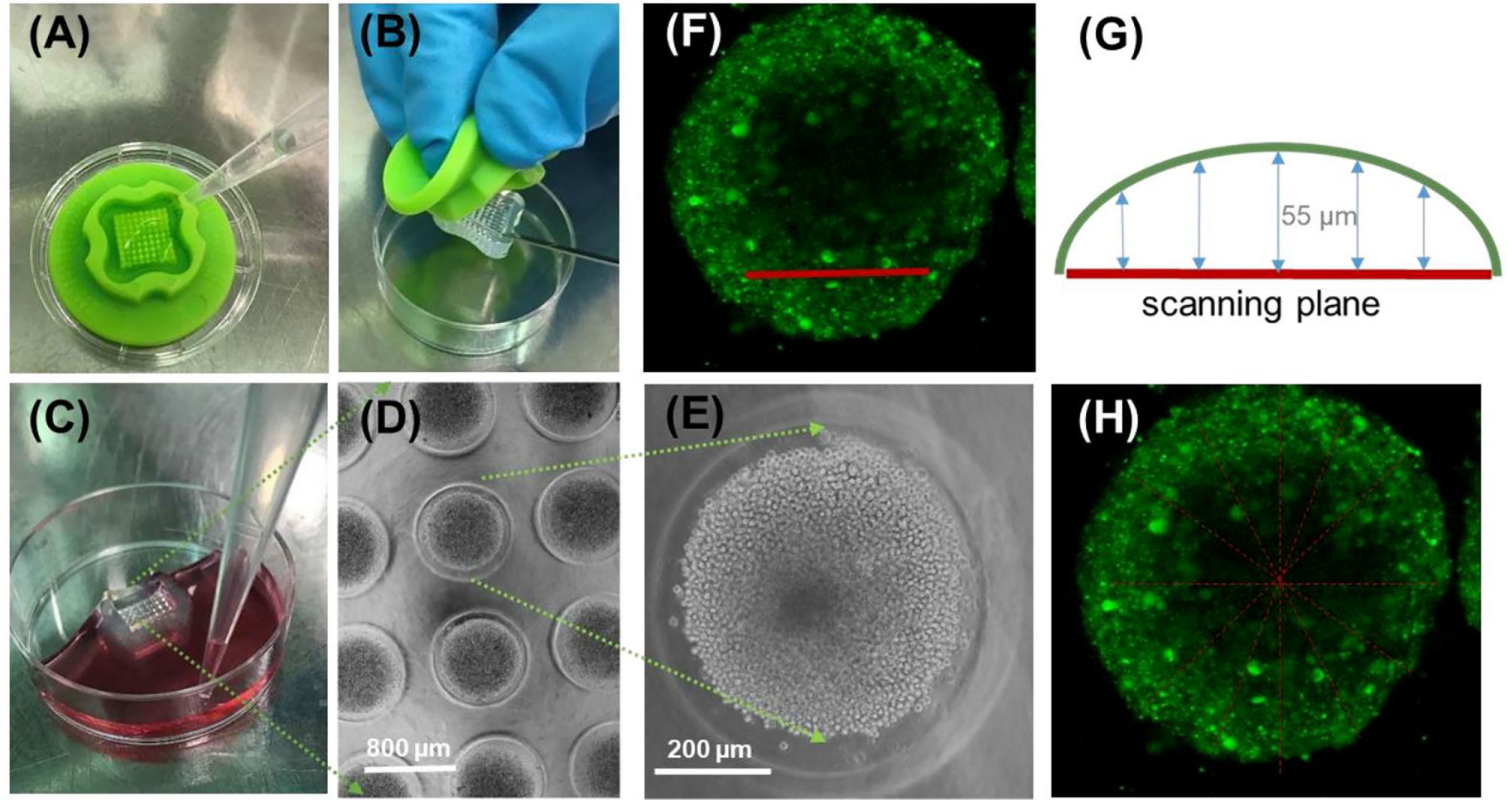
Schematic workflow using the micromolded nonadhesive agarose 3D Petri dish to evaluate *Cf*-peptide penetration ability to EBC-1 spheroid tissue-mimicking model. Imaging and analysis of spheroids **(A, B)**; 9 × 9-type silicone micromold used for casting agarose Petri dish **(C)**, equilibrating and adding complete cell medium. View of EBC-1 spheroids in agarose 3D Petri dish **(D)**, enlarged image of an individual EBC-1 spheroid **(E)**; **(D, E)** bright-field images were captured with an Olympus CX41 microscope [objective: ×4 **(D)** and ×20 **(E)**]. Side view of an EBC-1 spheroid with the position of scanning plane **(G)** during confocal laser scanning microscopy imaging (section z5, corresponding to z-section captured 55 µm from the surface) **(F)**. Position of lines during linescan of section z5 of two parallel spheroids (eight lines each) **(H)**.

In this study, we have employed a modified method based on (55). Briefly, micromolds for casting 3D Petri dishes (MicroTissues, Sigma, 5 × 7 array) were filled with molten agarose (2% (*w*/*v*) in PBS). The gelled agarose dishes were equilibrated with serum-free DMEM (2 ml/2 h, 37°C). Cells were seeded after incubation (1.8 × 10^4^ cell/µL in DMEM CM). Prior to seeding process, nuclei were stained with Hoechst 33342 solution (0.2 µM) for 30 min (**Figure 2**). Based on our previous work (55), the nuclear stain could not successfully penetrate into deeper layers of the spheroids (mostly localized at the spheroid surface), and towards the center of the spheroids the Hoechst 33342 signal was gradually decreasing. Stained cells were incubated in 2 ml DMEM CM for 48 h while cell-to-cell adhesion drives the aggregation and formation of spheroids. To monitor the condition of the spheroids, bright-field images were captured (Olympus CX41). After 48 h of incubation, spheroids were washed two times with fresh ICM DMEM and were treated with the *Cf*-Dhvar4 peptide (10 µM/2 ml ICM DMEM for 3 h). After the treatment, spheroids were washed two times with ICM DMEM and three times with PBS. Spheroids were fixed with 4% paraformaldehyde for 15 min (37°C) and were washed three times with PBS, and then harvested and transferred from the agarose micro-wells into µ-Slide 8-well uncoated Ibidi chambers for imaging (Zeiss LSM 710; ×10 dry objective (×10/0.45 M27)). The same excitation and emission wavelengths were used as for the 2D confocal microscopy imaging. Z-stack images were obtained by scanning the spheroids from the bottom of the spheroid with 11 µm distance between each scanning plane (**Figure 2**). Images were processed with ZEN 3.0 blue lite software. Line scan analysis was performed by NIH ImageJ software, two spheroids were analyzed (eight scans per spheroid, *n* = 16 in total). Line scans were carried out using greyscale images; grey value corresponds to the intensity of a given pixel on a scale of 0 to 255. As spheroids are of various shapes and sizes, the diameter of the scanned area (horizontal axis distance) was normalized to 1 for better comparison. To average the intensity values from line scans of spheroid sections of slightly different sizes and shape, all line scan lengths were normalized to 1, yielding normalized diameter. Calculation of average and SEM of multiple curves was carried out using the software OriginPro 2018.

### Intracellular Killing of Mycobacterium tuberculosis

Prior to experiment, MonoMac-6 cells were cultured in a 24-well plate for 24 h (2 × 10^5^ cells/1 ml medium well). Adherent cells were infected with *Mtb* H37Rv at a multiplicity of infection (MOI) of 10 for 4 h. Nonphagocytized extracellular bacteria were removed, and the culture was washed three times with serum-free RPMI. The infected monolayer was incubated for 1 day before antitubercular treatment. Infected cells were then treated with the compounds at 5, 10, and 50 μM concentrations. After 3 days, the treatment was repeated with fresh solution of the compounds for an additional 3 days. After washing steps to remove the compounds, infected cells were lysed with 2.5% sodium dodecyl sulfate solution. Ten times serial dilutions were then prepared from the lysates and plated onto BBL Löwenstein-Jensen tubes. After 4 weeks of incubation, the CFU of *Mtb* was enumerated, and the bacterial number was estimated and graphically presented. Experiments were repeated at least twice.

### Ethical Statement

Animal experiments were carried out in accordance with the guidelines of EU Directive 2010/63/EU and Hungarian laws and were approved by the Hungarian Scientific Ethical Committee on Animal Experimentation under the following protocol number: PE/EA/2569-4/2016.

### Mice, *Mtb* Infection and Therapy

For infection experiments, 6–8-week-old BALB/c female mice were kept in ventilated cages and allowed *ad libitum* access to water and to a standard pellet diet. Mice were infected with a mid-logarithmic culture of *Mycobacterium tuberculosis* H37Rv (10^6^ CFU/ml in PBS, 200 μl, i.p.). Two weeks after, therapy of 20 mg/kg bw of INH in 100 µl PBS (*per os*) and 30 mg/kg bw of INH-Dhvar4 in 100 µl PBS (*s.c.*) has started. Mice were treated twice a week for 5 weeks. Mice were monitored twice per day for the parameter attitude, locomotion, breathing, curiosity, nasal secretion, grooming, and dehydration. Mice that lost >20% body weight and had evidence of severe symptoms, such as scruffy coat, inactivity, loss of appetite, poor locomotion, or painful posture, were sacrificed before the termination of the experiments.

One week after the 10th treatment, mice were euthanized, and their organs were removed. To determine the viable bacteria, a portion of lung and spleen were homogenized in a tissue homogenizer using ceramic beads (MagNA Lyser Green Beads, MagnaLyser, Green Beads, Roche, Switzerland) in Bouillon broth (two times 60 s, 7,000 Hz), then 100 μl of the supernatant was plated onto BBL Löwenstein-Jensen Medium. CFU were counted after 4 weeks of incubation. For histopathological analysis, each animal’s lung, spleen, liver, and kidney were removed and fixed in 8% neutral buffered formalin for 24 h at room temperature before removing from the high-containment facility. Tissue specimens were dehydrated in a series of ethanol and xylene baths and embedded in paraffin wax. Sections (3–4 μm) were stained with hematoxylin and eosin (HE). For *in situ* visualization of the acid-fast bacilli, the Ziehl-Neelsen (ZN) staining method was applied on similar pretreated sections. Slides were analyzed in an Olympus BX53 microscope (Japan), and photomicrographs were obtained with an Olympus SC100 high-resolution digital color camera using the Olympus cellSens imaging software platform.

## Results

### Chemistry

For INH conjugation, representative cell-penetrating (CPP) and antimicrobial peptides (AMP) were chosen. These cationic, amphiphilic peptides are favored in the transportation of various cargos due to their high internalization rate. The used peptide series contains well-known CPPs such as penetratin, transportan, and Tat, frequently studied AMPs such as magainin and buforin, and designed analogs such as Dhvar4, Crot(1-9,38-42), and CM15. Also, a receptor-binding tuftsin analog (OT20), which was reported as a macrophage-targeting peptide, was also applied. **Table 1** shows the theoretical and experimental monoisotopic molecular masses of the peptide, measured on a high-resolution mass spectrometer. Mass spectra and RP-HPLC chromatograms are available at https://figshare.com/s/48a023a25068f7658adf, DOI: 10.6084/m9.figshare.16644901. Retention times observed on the analytical RP-HPLC chromatograms were also presented, which had a strong correlation to the overall hydrophobicity of the compounds. To study the internalization rate and intracellular localization, fluorescently labeled analogs were also synthesized. Note that the retention time of *Cf*-peptide are somewhat higher than unlabeled analogs, and in some cases, two peaks were detected due to the isomeric distribution of 5(6)-*Cf* molecule.

**Table 1:** Analytical characteristics of the peptides and peptide conjugates.

Peptide	Sequence	*M*_mo_ calc	*M*_mo_ meas^a^	Mass error (ppm)	Rt^b^
Penetratin	RQIKIWFQNRRMKWKK	2,244.3055	2,244.3057	0.15	6.1
Transportan	AGYLLGKINLKALAALAKKIL	2,180.4137	2,180.4130	0.66	10.6
Tat	YGRKKRRQRRR	1,557.9665	1,557.9665	0.02	0.9
Magainin	GIGKFLHSAKKFGKAFVGEIMNS	2,464.3413	2,464.3413	0.02	8.2
Buforin	RAGLQFPVGRVHRLLRK	2,001.2337	2,001.2336	0.10	6.6
Dhvar4	KRLFKKLLFSLRKY	1,838.1771	1,838.1766	0.54	6.9
Crot(1-9,38-42)	YKQCHKKGGKKGSG	1,503.8092	1,503.8092	0.02	0.8
CM15	KWKLFKKIGAVLKVL	1,769.1807	1,769.1800	0.72	8.4
OT20	TKPKGTKPKGTKPKGTKPKG	2,062.2739	2,062.2738	0.10	1.6
Melittin	GIGAVLKVLTTGLPALISWIKRKRQQ	2,844.7542	2,844.7530	1.20	10.6
*Cf-*Penetratin	*Cf-*AGYLLGKINLKALAALAKKIL	2,602.3527	2,602.3544	1.75	7.8
*Cf-*Transportan	*Cf-*GIGKFLHSAKKFGKAFVGEIMNS	2,538.4609	2,538.4631	2.21	12.2
*Cf-*Tat	*Cf-*YGRKKRRQRRR	1,916.0137	1,916.0137	0.03	4.6/5.0
*Cf-*Magainin	*Cf-*KRLFKKLLFSLRKY	2,822.3885	2,822.3907	2.16	9.6/9.7
*Cf-*Buforin	*Cf-*RAGLQFPVGRVHRLLRK	2,359.2809	2,359.2796	1.30	7.7/7.8
*Cf-*Dhvar4	*Cf-*RAGLQFPVGRVHRLLRK	2,196.2243	2,196.2250	0.66	9.0/9.1
*Cf-*Crot(1-9,38-42)	*Cf-*YKQCHKKGGKKGSG	1,861.8564	1,861.8567	0.33	5.5/5.8
*Cf-*CM15	*Cf-*KWKLFKKIGAVLKVL	2,127.2279	2,127.2270	0.94	9.6/9.7
*Cf-*OT20	*Cf-*TKPKGTKPKGTKPKGTKPKG	2,420.3211	2,420.3215	0.45	4.9/5.0
*Cf-*Melittin	*Cf-*GIGAVLKVLTTGLPALISWIKRKRQQ	3,202.8014	3,202.8028	1.37	12.2
**INH**-Penetratin	INH-RQIKIWFQNRRMKWKK	2,421.3593	2,421.3581	1.19	6.7
**INH**-Transportan	INH-AGYLLGKINLKALAALAKKIL	2,357.4675	2,357.4663	1.24	11.5
**INH-**Tat	INH-YGRKKRRQRRR	1,735.0178	1,735.0172	0.59	1.0
**INH**-Magainin	INH-GIGKFLHSAKKFGKAFVGEIMNS	2,641.3951	2,641.3947	0.40	9.0
**INH**-Buforin	INH-RAGLQFPVGRVHRLLRK	2,178.2875	2,178.2875	0.05	7.0
**INH**-Dhvar4	INH-KRLFKKLLFSLRKY	2,015.2309	2,015.2318	0.86	8.0
**INH**-Crot(1-9,38-42)	INH-YKQCHKKGGKKGSG	1,680.8630	1,680.8638	0.80	1.7
**INH**-CM15	INH-KWKLFKKIGAVLKVL	1,946.2345	1,946.2352	0.66	9.2
**INH**-OT20	INH-TKPKGTKPKGTKPKGTKPKG	2,239.3277	2,239.3273	0.38	4.1

C-terminus of the peptides was amidated. Note, that Cf was coupled as a 5(6) isomer which can be separated in some conjugates on the HPLC chromatograms.

a Exact molecular mass measured on a Thermo Scientific Q Exactive™ Focus Hybrid Quadrupole-Orbitrap™ Mass Spectrometer.

b Retention time on Waters Acquity UPLC BEH C18 (1.7 µm, 150 × 2.1 mm) column, flow rate: 0.3 ml/min, gradient: 2% B, 1 min; 2%–100% B, 16 min.

**Figure 3:**
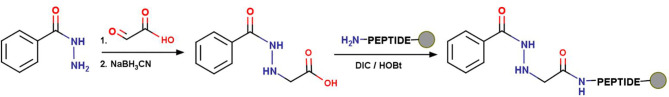
Coupling of isoniazid to peptides. First, INH was derivatized by glyoxylic acid, then reduced with NaBH_3_CN. The corresponding carboxylate was coupled to the peptides on solid phase using DIC/HOBt coupling reagents.

INH was coupled to the *N*-terminus of the peptides, using a previously described method (44). Resulted INH-peptide conjugates were then cleaved from the resin with TFA in the presence of scavengers (**Figure 3**).

Generally, after the cleavage and purification of synthetic peptides, TFA can remain in the lyophylisates. Residual TFA content can be deteriorative during the *in vivo* experiments; therefore, the removal of TFA and the replacement of TFA counter-ion to a less harmful acetate ion is strongly recommended. For that, an anion-exchange method was applied, which is described in the **Supplementary Information 1.1**).

**Figure 4:**
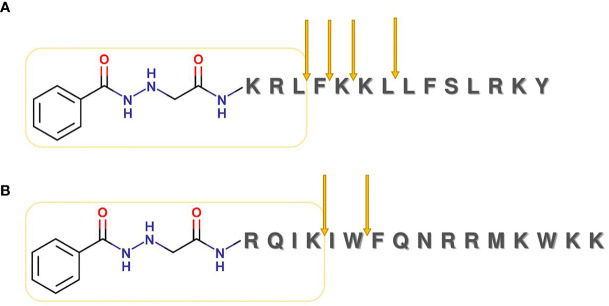
Lysosomal degradation of INH-Dhvar4 **(A)** and INH-penetratin **(B)** conjugate. Conjugates were incubated with rat liver homogenate, and samples from different time points were analyzed by LC-MS. For the detailed analysis (chromatograms and mass spectra), see **Supplementary Figure S1**.

Next, we examined the enzymatic stability of two peptide conjugates in rat liver lysosomal homogenate (see **Supplementary Information 2** for the method description). Peptides were dissolved at 0.025 µg/µl concentration then allowed to react with the lysosomal homogenate. Degradation of the conjugates was fast. After 30 min of incubation, the peaks of the intact conjugates almost disappeared from the chromatograms (**Supplementary Figure S1**). In the case of INH-Dhvar4, the most stable fragment was conjugated, which gave the main peak after 24 h of incubation, containing a tripeptide (KRL) covalently attached to INH (**Figure 4A**). In the case of INH-penetratin, the most stable fragment was a tetrapeptide (RQIK) with INH (**Figure 4B**).

### Internalization, Intracellular Localization, and Cell Surface Morphology

**Figure 5:**
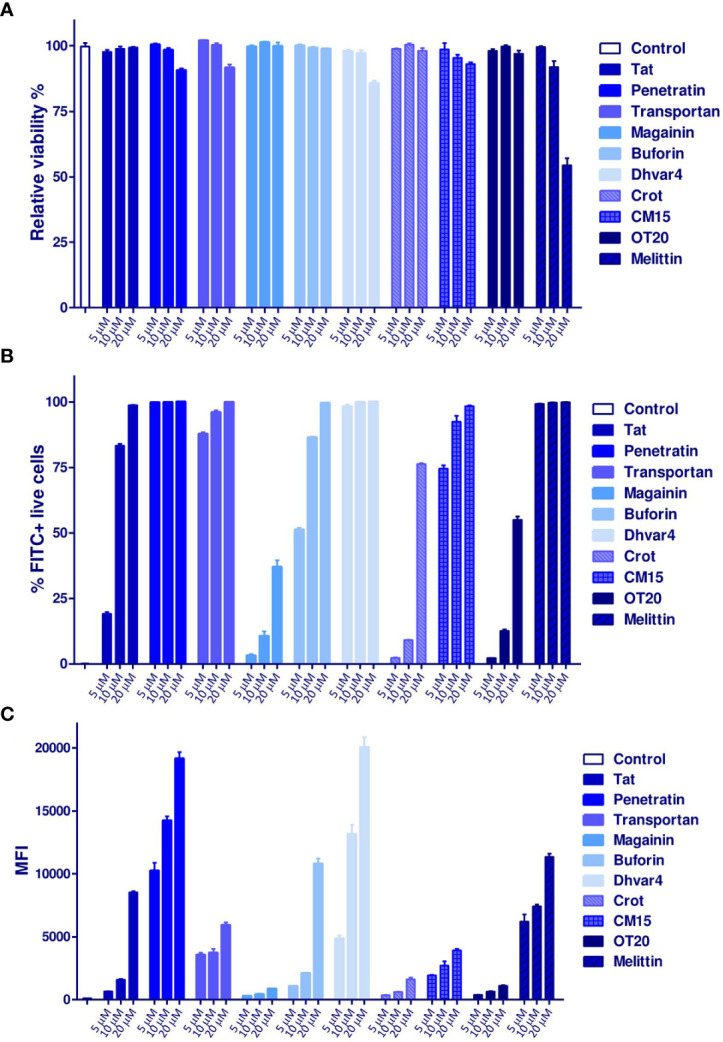
Internalization of *Cf*-labeled peptides to MonoMac-6 human monocytes. Cells were treated with the peptides for 2 h, then after trypsinization, the internalization rate was measured by a BD LSR II flow cytometer. **(A)** The relative viability of the cells compared with the medium-treated control cells. The percentage of FITC (*Cf*)-positive cells is presented in **(B)**, while the mean fluorescent intensity (MFI) is shown in **(C)**. Bars represent the mean of three parallels ± SEM. For analysis of statistical significance, unpaired *t*-test was used.

First, cellular uptake of MonoMac-6 human monocytes was studied. This cell line was utilized as a host for *Mtb* in the next set of experiments. Cells were treated with *Cf*-labeled peptides at 5, 10, and 20 µM concentrations for 2 h. In order to remove the surface-bound peptides, trypsin was added to the cells; then, the intracellular fluorescent intensity was measured by flow cytometry. At the studied concentration, none of the peptides showed more than 20% cytotoxicity (the relative viability was >80%), except melittin, where the cytotoxicity was almost 50% at the highest treatment concentration (**Figure 5A**). Highly efficient internalization was measured for the conjugates. The two most effective peptides were the Dhvar4 and penetratin (**Figures 5B, C** **)**.

**Figure 6:**
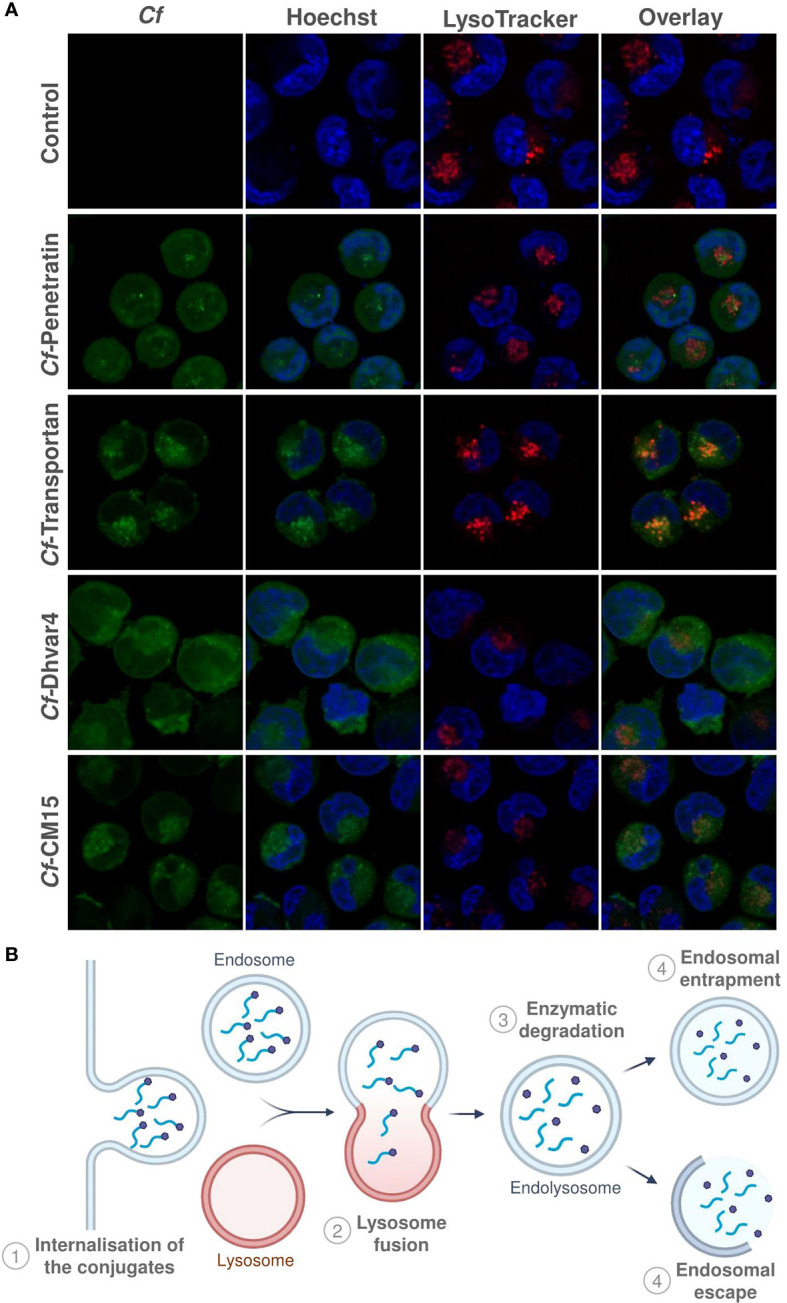
Intracellular localization of *Cf*-peptides. MonoMac-6 cells were treated with the peptides at 10 µM concentration for 2 h, then cells were captured by confocal laser scanning microscopy (Zeiss LSM-710 system) **(A)**. LysoTracker™ Deep Red was used for lysosome and Hoechst 33342 for nuclear staining. Schematic representation of internalization and endosomal entrapment/release processes is presented in **(B)**.

Parallel with flow cytometry measurements, confocal laser scanning microscopy images were also captured in order to assess qualitative information on intracellular localization. LysoTrackerTM Deep Red was used for lysosome and Hoechst 33342 for nuclear staining to distinguish subcellular localization of the *Cf*-peptide. The experiment was carried out after 2 h of incubation time, and representative images are presented in **Figure 6A**. As was expected, based on the results of flow cytometry measurements, all of the peptides were internalized efficiently. In the case of peptide transportan and CM15, fluorescent signals were observed mainly in lysosomal compartments (**Figure 6A**). Peptide Dhvar4 and penetratin could be imaged in the cytosol with almost no or low level of colocalization with lysosomal staining, which suggests that these peptides internalize and display a ubiquitous distribution in the cytosol. Considering all these data, it was presumed that the Dhvar4 and penetratin peptides enter the cells in a concentration-dependent manner by direct penetration or by endocytosis, followed by an endosomal release (**Figure 6B**). This was further proved by the effectivity of INH-Dhvar4 and INH-penetratin on intracellular bacteria. MonoMac-6 cells are excellent as host cell models; however, preparing them for confocal microscopy is quite challenging. We have optimized the cell preparation process (including the *Cf*-peptide treatments, fixation, staining protocol, incubation times, concentrations, washing steps, etc.). Our main goal was to achieve no cell morphology deformation, no staining differences in order to increase the confidence that *Cf*-peptide and lysosomal stain occupy the same or different structure. Colocalization of *Cf*-peptide and LysoTracker may be subjectively identified by the cooccurrence, the simple spatial overlap which combined the contribution of both signals (green and red) when the images of each signal are superimposed (merged, see **Supplementary Figure S3**). So, for example, colocalization of *Cf*-transportan and LysoTracker was apparent in vesicles that appear orange because of the combined contributions of green signal (*Cf*-transportan) and red fluorescence (LysoTracker). We have chosen a representative *Cf*-peptide set to quantify roughly the differences among peptide localization using greyscale analysis. The detailed process and graphs were presented in the **Supplementary Material**. We should point out, however, that this approach has its limitation, because our main goal was to visualize the differences and our experiments were mainly designed for qualitative rather than quantitative comparisons.

In addition to the internalization experiments, the surface morphology of INH-peptide-treated and fixed EBC-1 cells was also studied with atomic force microscopy (AFM) (**Supplementary Information 1.3**). High-resolution imaging of the cell surfaces was performed with a Flex-Axiom AFM system (Nanosurf, Liestal, Switzerland).The representative images and surface roughness of the native and treated cells are shown in **Supplementary Figure S2**. Mean *Rq* values are somewhat higher in the case of peptide and conjugate-treated cells, compared with untreated cells, but the differences do not suggest membrane disruption. As a result, we can conclude that membrane integrity maintained after the Dhvar4 and INH-Dhvar4 conjugate treatment.

### Penetration Ability on Bronchial Interface and 3D Spheroid Model

**Figure 7:**
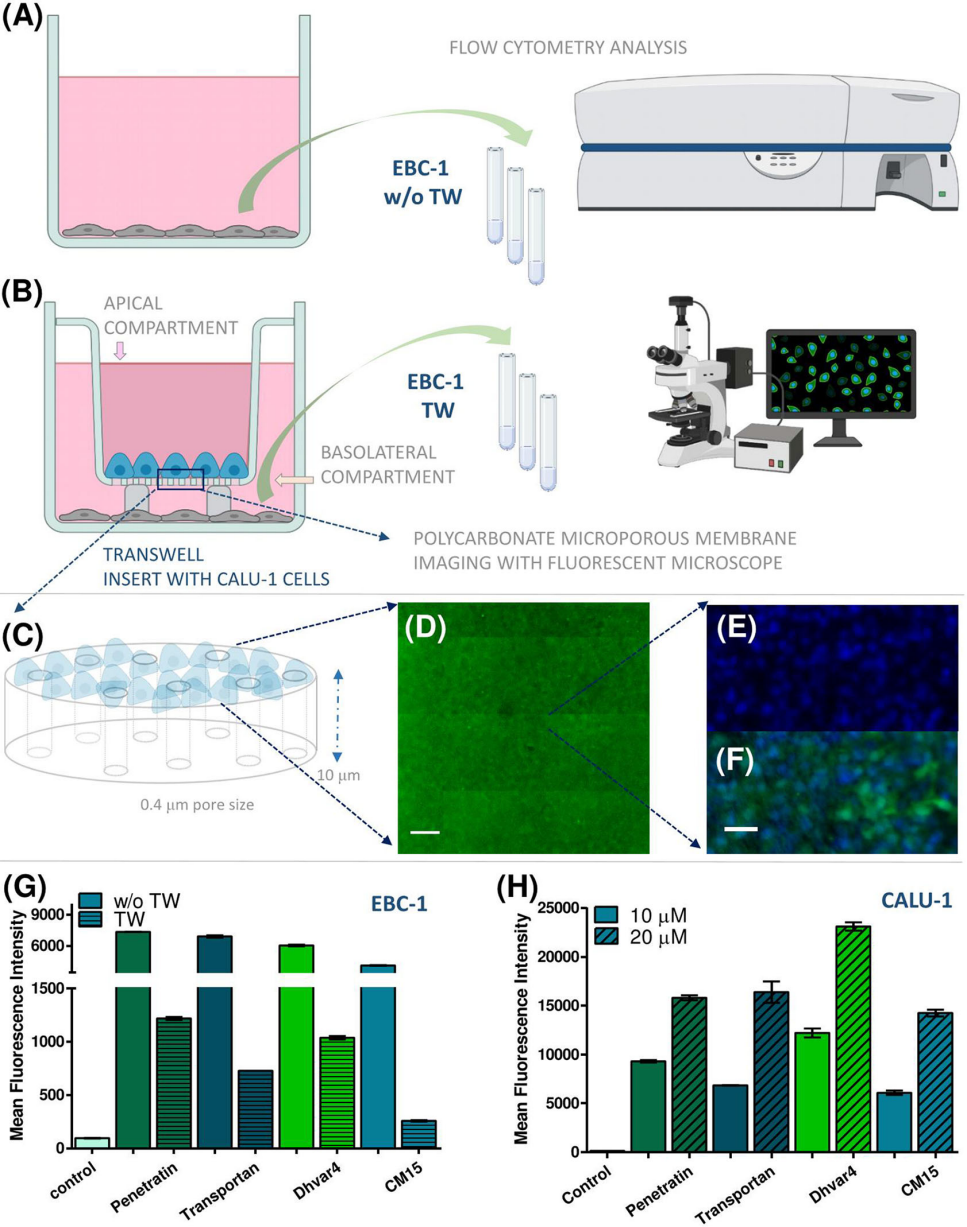
The penetration ability of *Cf*-peptides was determined on Transwell bronchial interface, and the penetration rates were quantified by flow cytometry. Schematic representation of the Transwell coculture arrangement with noncontact submerged monolayers as simple bronchial interface model containing CALU-1 and detector EBC-1 cells **(A, B)**. CALU-1 cells were seeded on the apical side of the TW insert, while EBC-1 cells were seeded on the bottom of the basolateral chamber (with or without Transwell inserts) **(A, B)**. Schematic enlarged features of the polycarbonate microporous membrane **(C)**. The confluence of CALU-1 monolayer was monitored with CellTracker Green, and after development of stable green fluorescence, cells were imaged *in situ*. Image acquisition was performed with Zeiss Axio Observer Z1 inverted epifluorescent microscope (×10 Plan Neofluar/×40 EC Plan-Neofluar objectives). The microscope was equipped with a Zeiss AxioCam MRm CCD camera and a Marzhauser SCAN-IM powered stage. For multifield mosaic image acquisition, stage positioning and focusing were controlled by Zeiss Axiovision 4.8 software. Images were processed using NIH ImageJ software. Scale bar represents 500 µm **(D)**. Enlarged section of the confluent monolayer of CALU-1 on the polycarbonate membrane. Nuclei of the cells were stained with Hoechst 33342 (blue) and *Cf* peptide (green). Scale bar represents 100 µm **(E, F)**. The flow cytometry analysis of detector EBC-1 cells with or w/o Transwell inserts, MFI values presented **(G)**. Cellular uptake of the *Cf*-peptides on CALU-1 cells compared by MFI values (treatment: 3 h, 10 and 20 μM) **(H)**.

First, we studied the penetration ability of the *Cf*-peptide on Transwell using submerged, cocultured noncontact monolayers of CALU-1 and EBC-1 cells as a simple *in vitro* bronchial interface model **(** **Figure 7**). *Cf* peptides were added to the CALU-1 containing apical chamber at 10 and 20 µM concentration and after 45 min or 3 h of incubation. EBC-1 cells from the basolateral chamber were stained and fixed prior to confocal microscopic analysis. As control experiments, treatments without TW were also carried out (**Figure 7G** **).** All four peptides were able to cross the CALU-1 monolayer, and they were internalized into EBC-1 cells. According to the green signal intensity, the internalization rates followed the same pattern as obtained in the cellular uptake studies (**Figure 5C**), namely, penetratin, and Dhvar4 have higher internalization rates (after crossing membrane and CALU-1 barrier) than the other peptides. Similar tendencies were observed after 3 h of incubation (data not shown).

**Figure 8:**
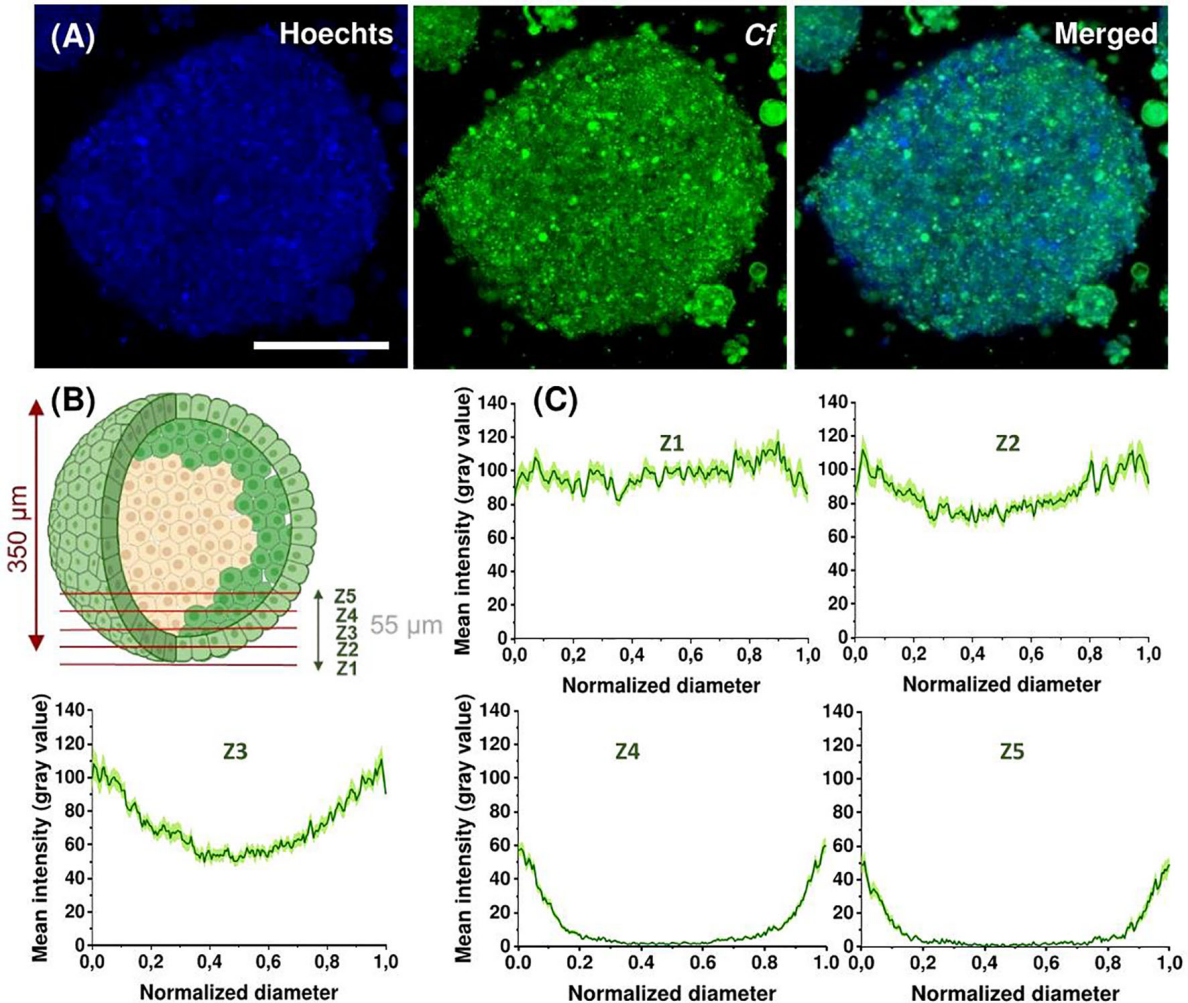
Representative confocal images of EBC-1 spheroids after 3 h treatment with 10 µM *Cf*-Dhvar4 (green). Nuclei were stained with Hoechst 33342 (blue). Z-stack images were obtained starting at the surface of the spheroid with 11 µm intervals for a total of 55 µm into the spheroid, the presented images are from the 22.6-µm (z2) depths. Scale bar represents 200 µm **(A)**. Schematic representation of z sections (z1–z5) **(B)**. Line scans of spheroids at different (z1–z5) depth, mean intensities are grey values (0–255) averaged from two parallel spheroids (eight line scans for each, total *n* = 16 line scans), error stripes correspond to SEM **(C)**. Imaging was performed by Zeiss LSM 710 system with a ×40 oil objective with the parameters: *Cf*-peptides *λ*ex = 488 nm, *λ*em = 541 nm, nuclei *λ*ex = 405 nm, *λ*em = 467 nm (Hoechst 33342).

Next, we assessed the penetration ability of *Cf*-Dhvar4 peptide on spheroids formed from EBC-1 cells. Cells were treated with the peptide for 3 h at 10 µM concentration. Nuclear staining with Hoechst 33342 was also applied. Fixed spheroids were visualized by confocal laser scanning microscopy. The EBC-1 spheroids had an average diameter of 350 and 400 nm, respectively (**Figure 8**). To visualize the penetration profile of the *Cf*-Dhvar4, we have carried out line scan analysis extracted from the images. Spheroids were scanned in the z-direction with a step size of 11 µm (z1–z5). The deepest z-section from the surface of a spheroid presented here is approximately 55 µm (z5). To have comparable fluorescent intensity values among the zones, normalized grey value data were standardized as the proportion of the mean normalized intensity in the peripheries (0%–100%). Towards the center of the spheroids, the *Cf* signal was gradually decreasing (**Figure 8**). Based on the presented results, we can conclude that *Cf*-Dhvar4 has fair penetration ability on EBC-1 spheroids.

### Antitubercular Effect on Extracellular and Intracellular *Mtb* Populations

Antibacterial effect against extracellular *Mtb* was determined using a conventional broth dilution method. The MIC value for INH was between 0.1 and 0.5 µM (0.01–0.07 mg/L), which is in accordance with literature data (56, 57). INH-peptide conjugates exhibited similar MIC values, namely 0.5–1.25 µM, which proved that INH maintained its activity in the conjugated form. This observation is in line with our previous results published recently (39, 44).

**Figure 9:**
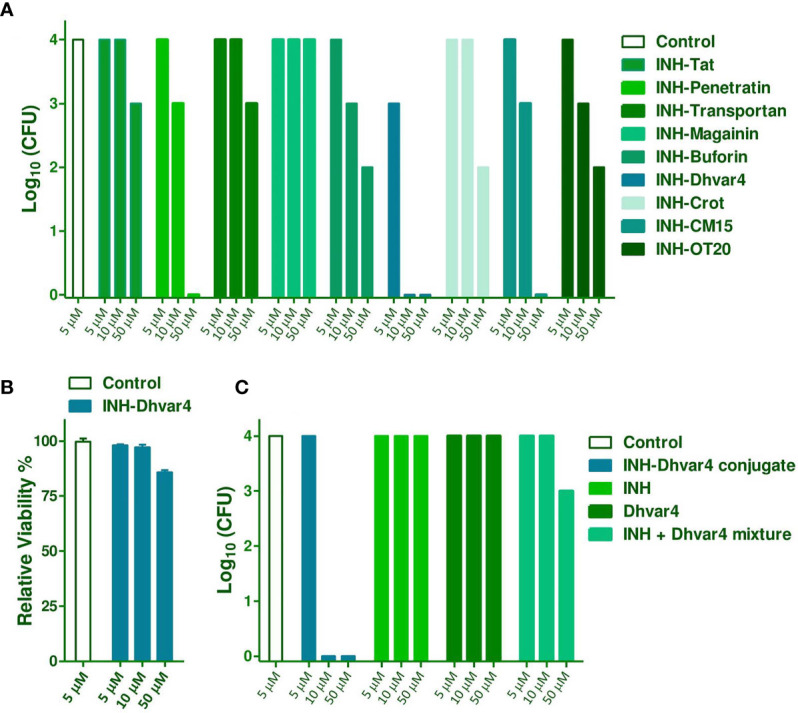
Intracellular killing efficacy of the INH-peptide conjugates was measured on *Mtb* H37Rv-infected MonoMac-6 cells **(A)**. Infected cells were treated with the conjugates at 5, 10, and 50 μM concentrations, two times for 3 + 3 days. After washing and SDS treatment, lysates were plated on Löwenstein-Jensen tubes and bacterial colonies were enumerated 4 weeks later. Cytotoxicity of INH-Dhvar4 to the host cell is presented in **(B)**. For a more detailed picture on the efficacy against intracellular *Mtb*, INH, Dhvar4 alone, and in mixture was compared with the INH-Dhvar4 conjugate **(C)**.

Intracellular killing activity of the INH-peptide conjugates was tested on *Mtb* H37Rv-infected MonoMac-6 human monocytes. Infected cells were treated two times for 3 + 3 days at 5, 10, and 50 µM concentrations. After washing and SDS lysis, Löwenstein–Jensen tubes were inoculated with the lysates, and bacterial colonies (CFU) were enumerated after 4 weeks of incubation. The most efficient compound was the INH-Dhvar4; therefore, this conjugate was studied in more detail. Cytotoxicity to the host cells was also measured (**Figure 9A**). The viability of MonoMac-6 cells, compared with medium-treated control cells, was >80% at the highest treatment concentration (50 µM) (**Figure 9B**).

Intracellular killing efficacy was further studied with different controls. INH itself was not effective against the intracellular form of *Mtb* at the studied concentration range, which is in line with previous observations (58, 59). Dhvar4 peptide itself was also not effective on the intracellular population of *Mtb*, but INH and Dhvar4 mixture showed a moderate antibacterial effect. When INH was covalently conjugated to the Dhvar4 peptide, significantly reduced bacterial growth was measured within the monocytes (**Figure 9C**). As low as 10 µM concentration was enough from the conjugate to be able to eliminate *Mtb* from the cells.

### Slow Progressing Murine TB Model

Taking together all *in vitro* data obtained for the conjugates, INH-Dhvar4 proved to be the optimal candidate for the *in vivo* evaluation.

**Figure 10:**
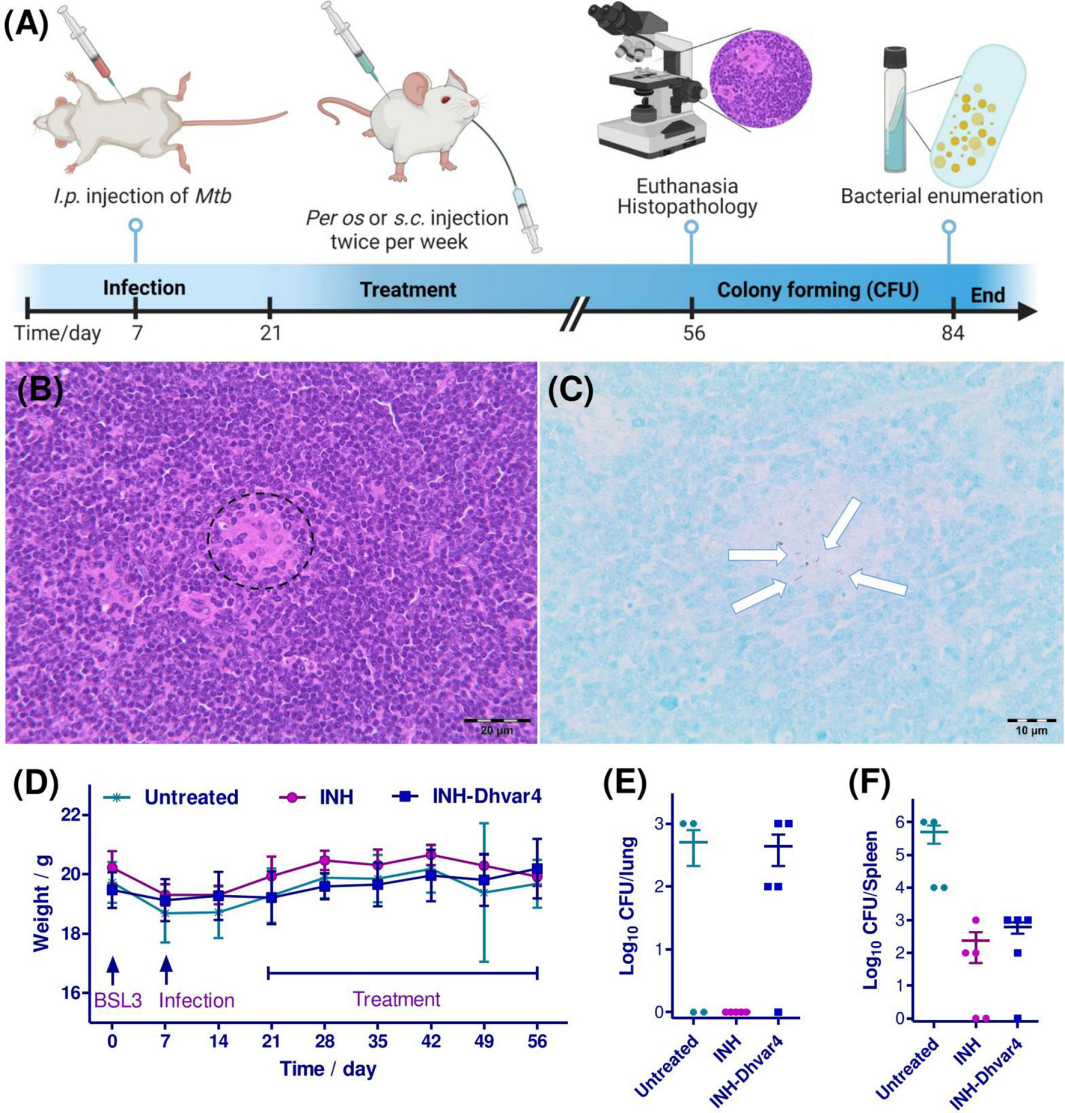
Timeline and results of the *in vivo* experiments. Mice were infected with *M. tuberculosis* H37Rv on day 7. After 2 weeks, mice were treated with INH (20 mg/kg bw *per os*) and INH-Dhvar4 conjugate (30 mg/kg *s.c.*) twice a week for 5 weeks **(A)**. Histopathological examination of the spleen of untreated control animals showed multifocal, small aggregates of macrophages in the white pulp [**(B)**, dashed circle], whereas Ziehl-Neelsen staining prepared from consecutive sections revealed the presence of rod-shaped, acid-fast bacteria within these lesions **(C)**, arrows], which clearly indicates *Mycobacterium tuberculosis* infection in the spleen. **(D)** The weight gain of the mice during the whole experiment. Each treatment group contained five animals, except for the control group, where the experiment ended up with four animals. One week after the 10th treatment, mice were euthanized, and the number of bacteria was enumerated from the tissue homogenates [**(E)**, lung; **(F)**, spleen].

In this study, a BALB/c murine model was applied with *i.p.* injection of 10^6^ CFU/ml *Mtb* bacteria (**Figure 10A**). This type of infection induces a course of slowly progressive and systemic disease and provides an opportunity for a prolonged experimental setup. The well-being of the animals, monitored by the body weight, showed an unbiased gain till the last week of the experiment (**Figure 10D**). After euthanasia, organ homogenates were cultured on Löwenstein-Jensen media and CFU were counted. Beside the lung, mice contained a high number of *Mtb* within the spleen, leading to the progressive systemic spread of the bacteria. In histological sections of the spleen of untreated control animals, rod-shaped acid-fast bacteria were observed, indicating the successful experimental design to model TB (**Figures 10B, C** **)**. INH was administered *per os* (20 mg/kg bw), while INH-Dhvar4 conjugate was administered *s.c*. (30 mg/kg bw). Note that the INH content of the conjugate was only 2 mg. Each treatment group contained five animals, but after the seventh week of the experiment, one mouse from the untreated (but infected) control group was euthanized. The measured weight loss indicated this action (4 data points on the CFU/organ panels for the untreated control group). Clearance of *Mtb* from the lung was experienced after INH treatment (**Figure 10E**), while only a moderate decrease was observed in the spleen (**Figure 10F**). The same decrease in the number of *Mtb* counted from the spleen homogenates was observed for the INH-Dhvar4 conjugate, which contains only 1/10 of INH compared with the orally administered drug. The progress of the infection is extremely a complex process *in vivo*, and it is affected by the relative density of bacterial subpopulations, tissue microenvironments and consequently the respective susceptibility of the individual animals to therapy. The aforementioned factors can result in the heterogeneity of the efficacy data. Our murine model was intended to be used as a slow progress one. The drawback of this model is that some animals had higher spleen and/or lung bacterial loads to resist the activity of the conjugate and to achieve the sterile cure.

## Discussion

The development of new, better-acting antituberculars is challenging because the compound should be effective against a variety of *Mtb* strains residing in different tissue environments. In this paper, a road map of experimental approaches is suggested to characterize a compound by its antitubercular effect, lysosomal stability, internalization to host phagocytes, penetration through tissue models, the intracellular killing of *Mtb*, and *in vivo* antitubercular activity in a murine model of TB. As a model compound to investigate this experimental road map, INH and its peptide conjugates were selected because of their different molecular profile, penetration ability, and intracellular activity.

**Figure 11:**
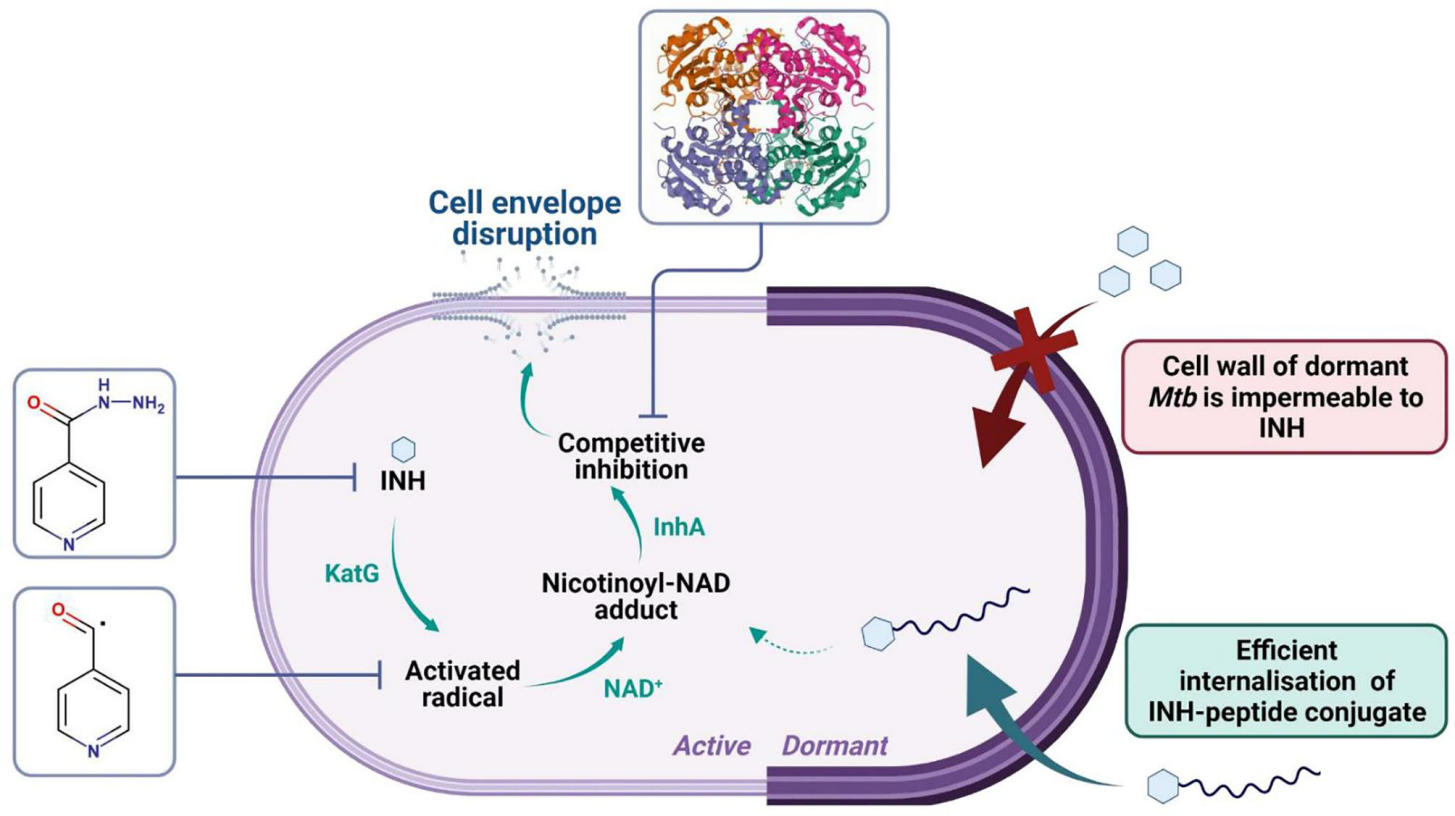
Cell wall alteration of dormant *Mtb* inhibits the internalization and antitubercular activity of INH. Isoniazid penetrates the cell wall of actively growing *Mtb*. The prodrug is activated by the mycobacterial KatG enzyme, which converts INH to a nicotinoyl radical. The activated radical forms a covalent adduct with NAD^+^ or NADP^+^. The INH-NAD adduct acts as a tight-binding competitive inhibitor of InhA (PDB ID: 4TRN). InhA (enoyl acyl carrier protein reductase) catalyzes a fatty acid elongation cycle in the type II fatty acid synthase (FAS-II) system, which is involved in the biosynthesis of mycolic acid. The inhibition of the synthesis of this essential cell wall component leads to membrane disruption and, as a consequence, death of the bacterium. INH is highly active against replicating but not against dormant or near-dormant bacteria. During dormancy, *Mtb* is shielded by an extremely robust cell wall, which is impermeable to INH and most drugs. With the help of peptide conjugation, efficient cell penetration of dormant, intracellular *Mtb* was observed.

INH is a prodrug, activated by the mycobacterial KatG enzyme (EC:1.11.1.21) (60). The activated radical forms INH-NAD adduct that binds to the active site of InhA (EC:1.3.1.9, PDB ID: 4TRN). InhA plays an important role in the biosynthesis of mycolic acid (61). Inhibition of this essential cell wall component is the main mechanism of action for INH (57, 62). In humans, INH is metabolized *via N*-acetylation of its hydrazine functionality by NAT2 enzyme (E.C. 2.3.1.5), which is followed by renal extraction (63, 64) (**Figure 11**). Drug-induced liver injury is the main adverse effect of INH leading to poor compliance and interruption of the treatment (65).

Cell wall alteration is the main attribute of *Mtb* in latent infection. The intracellular, dormant *Mtb* bacilli become phenotypically resistant to most drugs and almost unreachable to antitubercular therapy (56). Reduced drug uptake is the major mechanism responsible for drug resistance in TB therefore, enhancing the internalization rate to *Mtb*-exposed phagocytes and infected human granuloma has a great potential to develop better-acting antitubercular drugs.

Host-directed therapies utilizing cell-penetrating peptides have been suggested to treat diseases where the causative agent can remain in the host phagocytes (66). In this study, a set of representative cationic, amphiphilic cell-penetrating and antimicrobial peptides was applied as carrier moieties for Isoniazid. Well-known CPPs (penetratin, transportan, and Tat) together with frequently studied AMPs (magainin, buforin), as well as designed analogs [Dhvar4, Crot(1-9,38-42), CM15] and a macrophage-binding Tuftsin analog (67–69) were conjugated covalently with INH using a previously described method (44).

All of the used peptides possess the common feature of containing several positively charged amino acids (Lys, Arg) but differ in hydrophobicity and membrane affinity. These features strongly influence the internalization and intracellular activity of the compounds. The interaction of INH-peptide conjugates with lipid monolayers was the topic of our previous work (70). As a result, we could conclude that the degree of penetration into a TB membrane model, consisting of DPPC+mycolic acid (3:1), was the highest for INH-transportan, INH-CM15, INH-magainin, and INH-Dhvar4 conjugates. Somewhat lower penetration was observed for INH-penetratin. The membrane affinity order almost followed the calculated hydrophobicity, namely transportan, CM15, magainin, and Dhvar4 were the most hydrophobic compounds in the given set. The mycolic acid content provides a negatively charged membrane layer that is favored by the cationic peptide conjugates, and long alkyl chains reduce the regular close packing of the DPPC, allowing good penetration of the conjugates. Structural changes induced by the presence of negatively charged bacterial membrane were frequently observed for AMP and CPP peptides and their conjugates (71–74), which led to a more complex view on the intracellular effect of such conjugates.

To elucidate the *in vitro* penetration ability of the peptides, Transwell measurements using submerged, cocultured noncontact monolayers as a simple bronchial interface model was performed. This setup provided a remarkable difference in the trafficking pattern between *Cf*-peptide on the interface model mimicking lower respiratory tract. To compare the penetration and internalization capacity of the delivery peptides, we have employed flow cytometry analysis as an examination of differences, but we need to emphasize that these observations can be considered qualitative information. Furthermore, using 3D spheroid EBC-1 cultures for mimicking lung tissue *in vitro*, we have shown that Dhvar4 has fair penetration ability and can be further used as a drug delivery peptide. We have also demonstrated that agarose based spheroid system is a simple assay for comparative penetration studies using confocal laser scanning microscopy images with line-scan analysis to quantify differences.

Investigation of the internalization mechanism is essential for the development of drug compounds against intracellular pathogens. Their composition, size, total charge, and conformation in combination with cell membrane properties rule the internalization processes. Endocytosis, an energy-dependent active type of internalization, is a complex process composed of several steps [detailed in (75)]. The direct membrane translocation, a single-step passive internalization process, can be employed by delivery peptides as an alternative to endocytosis. We assume that the success of INH-Dhvar4 conjugate against intracellular *Mtb* is based on the efficient internalization and distribution within the cytosol (**Figures 5**, **6**).

Taken together, using this experimental setup as an assay platform, we significantly improved the evaluation process of drugs and drug conjugates. The applied *in vitro* MIC determination, selectivity and localization studies to elucidate efficacy and specificity, infected host model, coculture interface system on cell monolayers, and tissue-mimicking spheroids were suitable assays in order to find the most promising constructs for *in vivo* studies and for the rational design of delivery peptides targeting intracellular *Mtb* with superior tissue penetration ability. Prior to *in vivo* efficacy test, we have followed the recent principles of substituting animal toxicity experiments: (i) applying tissue-mimicking platform; (ii) interface assay; and (iii) using already available data. Employing these, it is possible to estimate the safe treatment dosages for the animals in the efficacy experiment. Therefore, in our study, we did not employ the conventional path for the *in vivo* toxicity study in order to decrease the number of animals, while remaining consistent to acquire valid results. There are similar antibacterial peptides with well-documented LD_50_ values and we could follow the 3R principle. Our focus in this study was to propose an assay platform rather than a drug candidate.

## Data Availability Statement

The datasets presented in this study can be found in online repositories. The names of the repository/repositories and accession number(s) can be found below: Figshare (https://figshare.com/s/48a023a25068f7658adf).

## Ethics Statement

The animal study was reviewed and approved by the Hungarian Scientific Ethical Committee on Animal Experimentation. Protocol number: PE/EA/2569-4/2016.

## Author Contributions

Conceptualization: SB and KH. Synthesis and chemical characterization of the peptides and conjugates: KH. Design, preparation of the *in vitro* monolayer-based methods, analysis—interpretation of the results: SB and KH. Work with extracellular and intracellular mycobacterial cultures: SB, KH, ZS, BP, and JH. Design, preparation, and analysis of the 3D spheroid: SB and BB-K. Design, preparation, and analysis of the Transwell experiment: SB. Design, preparation, and analysis of the animal experiments: SB, KH, KF, and BP. Histopathology and interpretation: GB. *Ex vivo* membrane integrity studies and analysis: GG and ÉK. Confocal microscopy image acquisition and analysis: BB-K and SB. Writing the manuscript: KH and SB. The manuscript was written through the contributions of all authors. All authors have given approval to the final version of the manuscript.

## Funding

This work was completed in the ELTE Thematic Excellence Programme 2020 supported by the National Research, Development and Innovation Office—TKP2020-IKA-05. KH thanks the Hungarian Academy of Sciences for the Premium Post-Doctoral Fellowship support. This work was supported by the National Research, Development and Innovation Office, Hungary (grants: VEKOP-2.3.3-15-2017-00020). SB, KH, BP and JH thank for the support of grant EFOP-1.8.0-VEKOP-17-2017-00001. We are grateful for the ELTE Thematic Excellence Programme (Szint+) and the 2018-1.2.1-NKP-2018-00005 project (under the 2018-1.2.1-NKP funding scheme) provided by the Hungarian Ministry for Innovation and Technology and for the National Research, Development and Innovation Office, Hungary.

## Conflict of Interest

The authors declare that the research was conducted in the absence of any commercial or financial relationships that could be construed as a potential conflict of interest.

## Publisher’s Note

All claims expressed in this article are solely those of the authors and do not necessarily represent those of their affiliated organizations, or those of the publisher, the editors and the reviewers. Any product that may be evaluated in this article, or claim that may be made by its manufacturer, is not guaranteed or endorsed by the publisher.
