# Supplementary material for: Novel Assay Platform to Evaluate Intracellular Killing of Mycobacterium tuberculosis: In Vitro and In Vivo Validation
Source: Front Immunol. 2021 Nov 12;12:750496. doi: 10.3389/fimmu.2021.750496 (PMC8632718; doi:10.3389/fimmu.2021.750496)
Supplement: Supplementary file 1 [file DataSheet_1.pdf]

Supplementary Material

1 Supplementary Data

1.1 Counter Ion Replacement and the Determination of Fluorine Content

For the *in vivo* measurements, toxic TFA from the lyophilisate of INH-Dhvar4 was removed by acetate-exchange using Amberlit IRA-400 (20-50 mesh, Cl^- form) anion-exchange resin. First, 5g resin was washed with water, then treated with 30 mL 0.2 M NaOH for 5 min, neutralized with water, and treated with 30 mL 20 v/v% acetic acid (5 min). After washing with water, the resin was mixed with the peptide solution (100 mg / 10 ml of water). The peptide-resin suspension was stirred for 1 h, then filtered and washed with 20 v/v% acetic acid and water. After freeze-drying, peptides were analysed by LC-MS and amino acid analysis. Amino acid analysis revealed that the peptide content of INH-Dhvar4 was 48 ± 2%. To calculate the anion-exchange rate, the Fluorine content of the peptides was determined by using a modified Schöniger method [1]. The exchange rate was calculated by dividing the percentage of the Fluorine content of acetate-exchanged peptide by the percentage of the Fluorine content of the original peptide.

1.2 Lysosomal degradation studies

Enzymatic stability of INH-Dhvar4 and INH-Penetratin conjugates were assayed in rat liver lysosomal homogenate (protein content was 16.6 µg/µl). Peptides were dissolved in 0.2 M Sodium acetate buffer (pH=5.03) at 0.025 µg/µl concentration and added to the lysosomal homogenate to reach 1 : 1 protein / peptide ratio (37°C, 600 rpm vortex). At different time points (5 min, 30 min, 1 h, 6 h, 24 h) the reaction was stopped by the addition of 5 µl formic acid. Sampled were then measured by LC-MS using a Thermo Scientific Q Exactive Focus Hybrid Quadrupole-Orbitrap Mass Spectrometer (**Suppl. Figure 1**). For chromatography, a Waters Acquity UPLC BEH C18 (1.7 µ, 150 × 2.1 mm) column was used with a flow rate of 300 µL/min applying a gradient elution from 2% to 100% of eluent B.

(A)

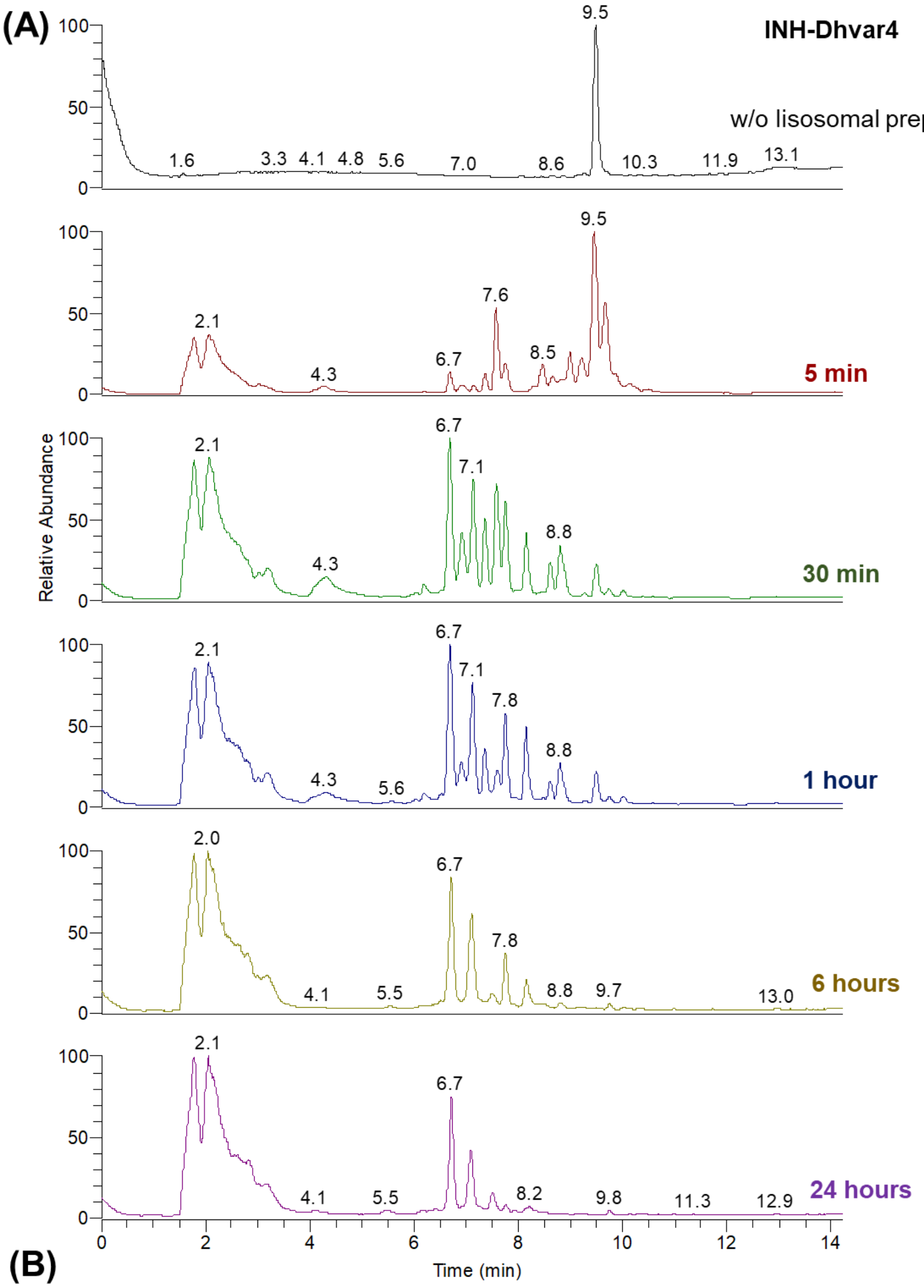

(B)

Rt /min	M monoisotopic	Fragment/peptide
9.5	2421.3618	**INH**-KRLFKKLLFSLRKY
6.7	592.3447	**INH**-KRL
7.1	995.6036	**INH**-KRLFKKL
7.8	867.5085	**INH**-KRLFK
8.8	739.4137	**INH**-KRLF

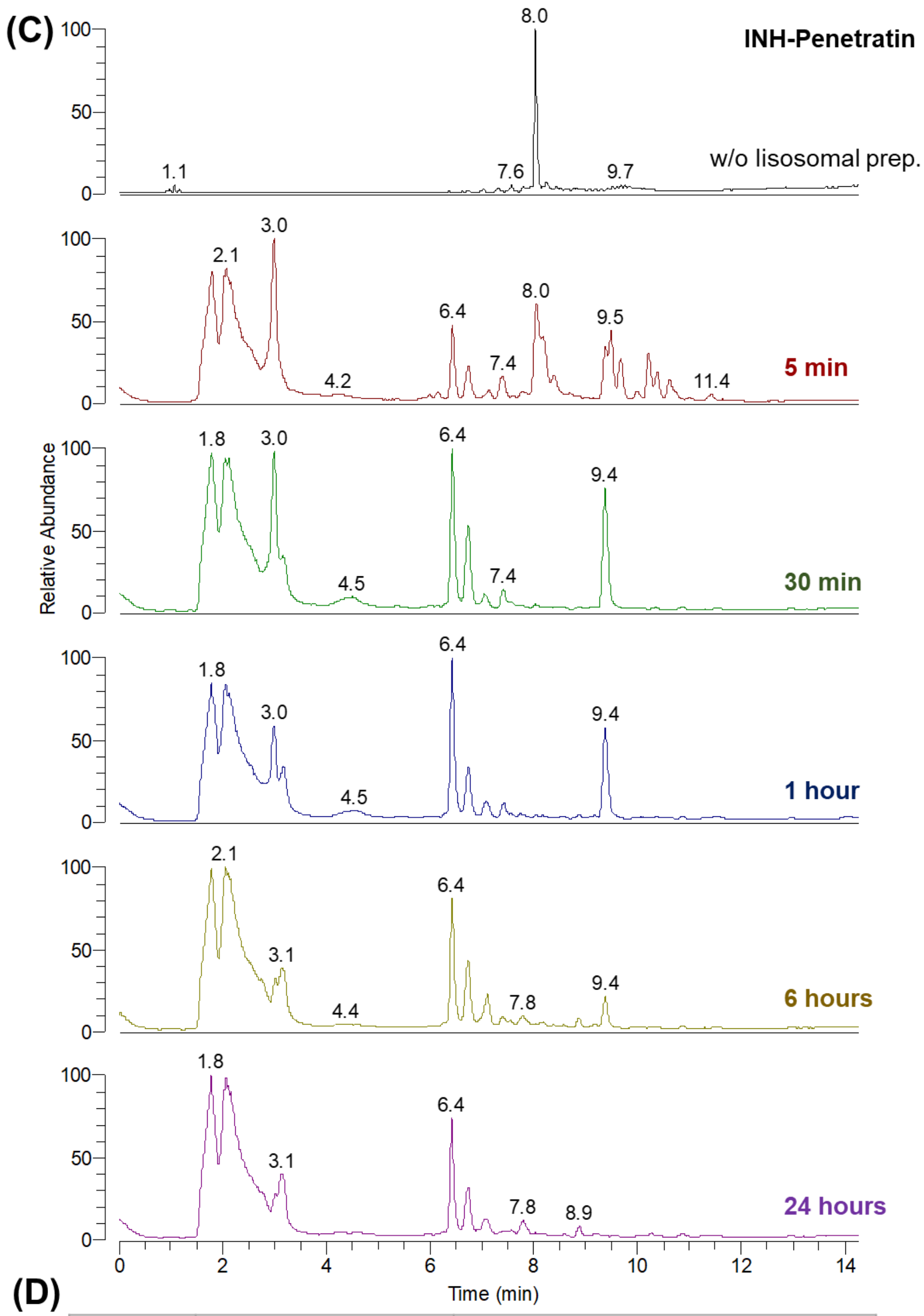

(D)

Rt /min	M monoisotopic	Fragment/peptide
8.0	2421.3618	**INH**-RQIKIWFQNRRMKWKK
6.4	720.4032	**INH**-RQIK
9.4	317.1738	IW

Supplementary Figure 1. Lysosomal degradation of INH-Dhvar4 (**A,B**) and INH-Penetratin (**C,D**) conjugate. LC-MS analysis of the samples after different reaction times: chromatograms (**A,C**) and the corresponding masses (**B, D**).

1.3 Membrane integrity evaluation by atomic force microscopy (AFM)

Prior to AFM analysis the EBC-1 cells were seeded in DMEM CM medium to 24-well cell culture plates on cover glasses (thickness 1, Assistant, Karl Hecht GmbH, Sondheim/Rhön, Germany) 24 hrs before the experiment (10^5 cells / 1 mL / well). Cells were treated with Dhvar4 and INH-Dhvar4 for 3 hrs at 10 μM (37°C, 5% CO_2). Control cells were incubated with medium. After the treatment, cells were washed two times with ICM and three times with PBS. Cells then were fixed with 4% glutaraldehyde (dissolved in PBS) for ON at 4°C. After fixation, cells were washed two times with PBS and two times with three distilled water.

Surface morphology of the fixed EBC-1 cells was investigated with atomic force microscopy (AFM). High resolution imaging of the surfaces were performed with a Flex-Axiom AFM system (Nanosurf, Liestal, Switzerland) operating in dynamic mode utilizing soft-tapping cantilevers (Tap150-G, BudgetSensors) with nominal force constant of 5 N/m. The recorded images were evaluated with the Gwyddion software. Representative images of the native and treated cells are shown in **Suppl. Figure 2A,B,C**.

Two cut-off frequencies were chosen with real-space wavelengths of 1 μm and 200 nm. Example line-profiles and their corresponding roughness profiles at the selected cut-off frequencies are shown in **Suppl. Figure 2D,E,F.** Scale dependent surface roughness of the cells was determined according to Antonio et al [2]. Mean Rq values are somewhat higher in the peptide and conjugate treated cells, but the differences not suggesting membrane disruption.

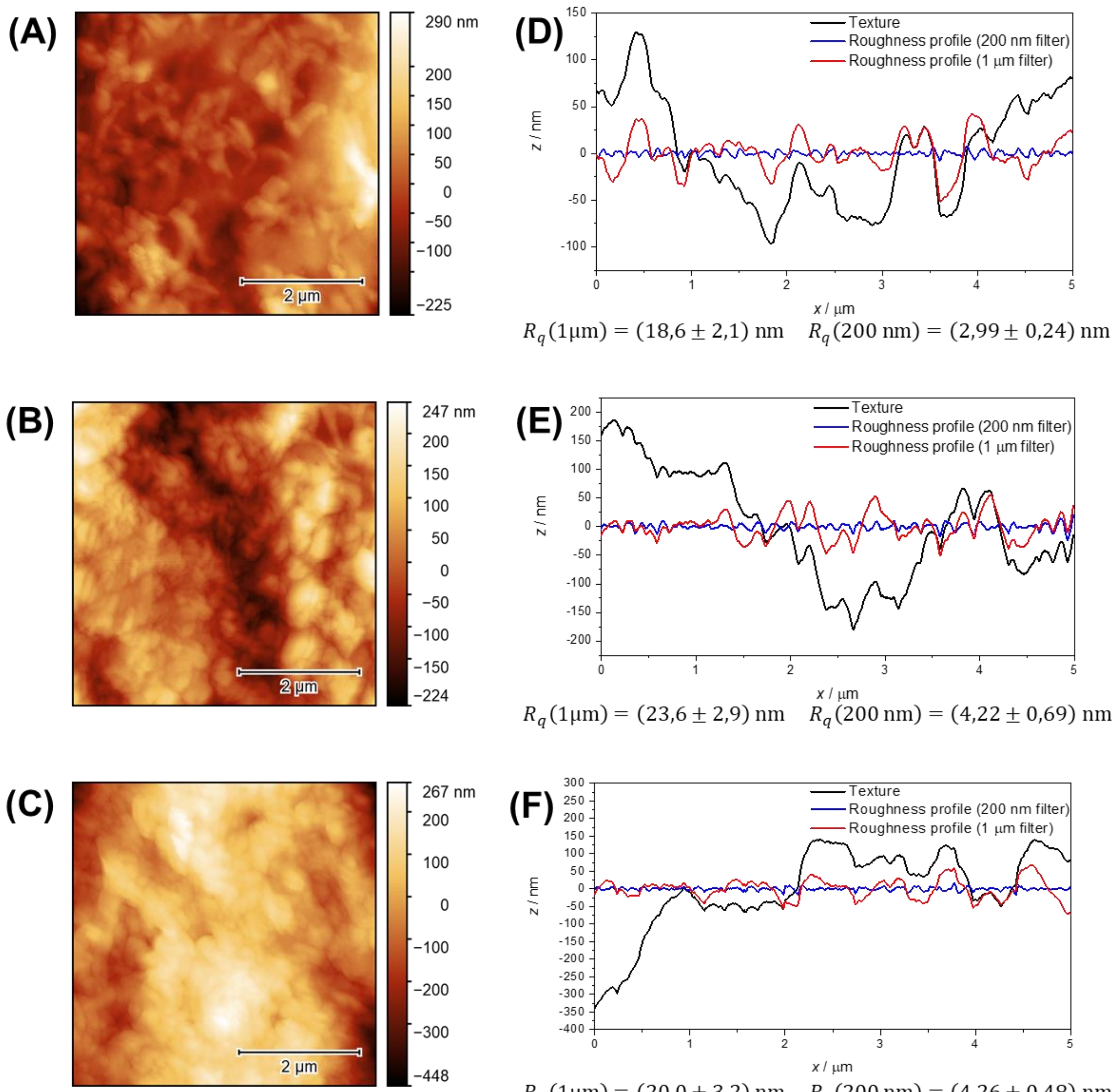

2

Supplementary Figure 2. High resolution morphology images of EBC-1 cells treated with medium (**A**), Dhvar4 (**B)** and INH-Dhvar4 conjugate (**C**). The apex area of the cells was selected for imaging with a scan area of 5 μm × 5 μm (512 × 512 pixel resolution), where the cellular curvature has the smallest distortion effect on the recorded height profiles. Panel **D, E** and **F** represent characteristic cross-section and roughness profiles with the mean and ± Sd of root mean square (*Rq*) values based on 30 profiles.

1.4. Intracellular localization of *Cf*-peptides using confocal laser scanning microscopy

We have visualized the intracellular localization of the *Cf*-peptides using confocal laser scanning microscopy at the 10 μM *Cf*-peptide concentration (based on flow cytometry profile), and MonoMac6 cells were incubated for 2 hrs. Representative images are presented in **Suppl. Figure 3**. To quantify

the visible differences among localization profiles of the *Cf*-peptides, we applied greyscale analysis of the images and the evaluation was carried out on a representative peptide set. Fixed MonoMac-6 cells were studied and the treatment, the staining and fixation process were carefully optimized to assess comparable greyscale values corresponding to green (intensity of *Cf*) and red (intensity of LysoTracker dye) signals. Laser intensity values were identical in case of all *Cf*-peptides. ZEN 3.0 blue lite software was used for image processing and grey scale values were extracted using NIH ImageJ software's Plot Profile application.

Cf-Transportan mainly accumulated in lysosomal compartments (based on lysosomal staining) (**Suppl. Figure 3A**.). Co-localization with the lysosomal staining can indicate vesicular transport involved in the uptake of *Cf*-peptides (**Suppl. Figure 3A**, upper part image with white arrows). For the *Cf*-Transportan the green and the red signals are of roughly similar intensity, and this similarity is most expressed on the areas where the signal (greyscale value) is high (**Suppl. Figure 3A**, lower part graph). The *Cf* signal only partially co-localizes with the cytoplasmic area. No co-localization with the nuclei staining can be observed, proved also by the line scan analysis of the representative cells (presented with low Hoechts intensity).

Based on the greyscale analysis, there is no vesicular transport involved in the uptake of the *Cf*-Dhvar4 (no direct co-localization with lysosomes, (**Suppl. Figure 3B,** upper part image and lower part graph). As it is shown on the graph, the green signal as a function of distance (pixel) and intensity, is high on areas corresponding to cytoplasm. At the same time, on those areas the red signal is negligible. These numerical data therefore show close correlation with the empirical observation we deduce from the captured image (**Suppl. Figure 3B**, upper part images and lower part enlargement)), this *Cf*-peptide internalizes and displays a ubiquitous distribution in the cytosol.

According to the fluorescent signals *Cf*-Dhvar4 is mainly localized in cytoplasm, while peptide *Cf*-CM15 is also accumulated in the cytosol and partially co-localized with the lysosomal staining and shows a different pattern (**Suppl. Figure 3C**, upper part images, lower part image with white arrows and graph). If the *Cf*-peptide internalize into the lysosome then the green and red signals co-localize (exhibit similar intensity in the function of the normalized distance), when the *Cf*-peptide is in the cytoplasm, then the graphs of the signals show different intensity.

We have also evaluated the co-localization using ImageJ JACoP [3, 4]. It was convenient to use, because we used the merged images for identification of the regions of interest. The calculated values presented the similar tendencies that was obtained from the greyscale analysis (**Suppl. Figure 3)**. The Pearson's coefficient value for *Cf*-Transpotan is 0.823, which represented strong correlation. In the case of *Cf*-Dhvar4 the coefficient value (0.249) suggested small correlation, therefore cytoplasmic occupation was detected mainly. Moderate degree was resulted for *Cf*-CM15 and *Cf*-Penetratin (0.358 and 0.490, respectively).

We should point out, however, that each approach (greyscale analysis and JACop) has its limitation and we have mainly designed our experiments for qualitative rather than quantitative comparisons.

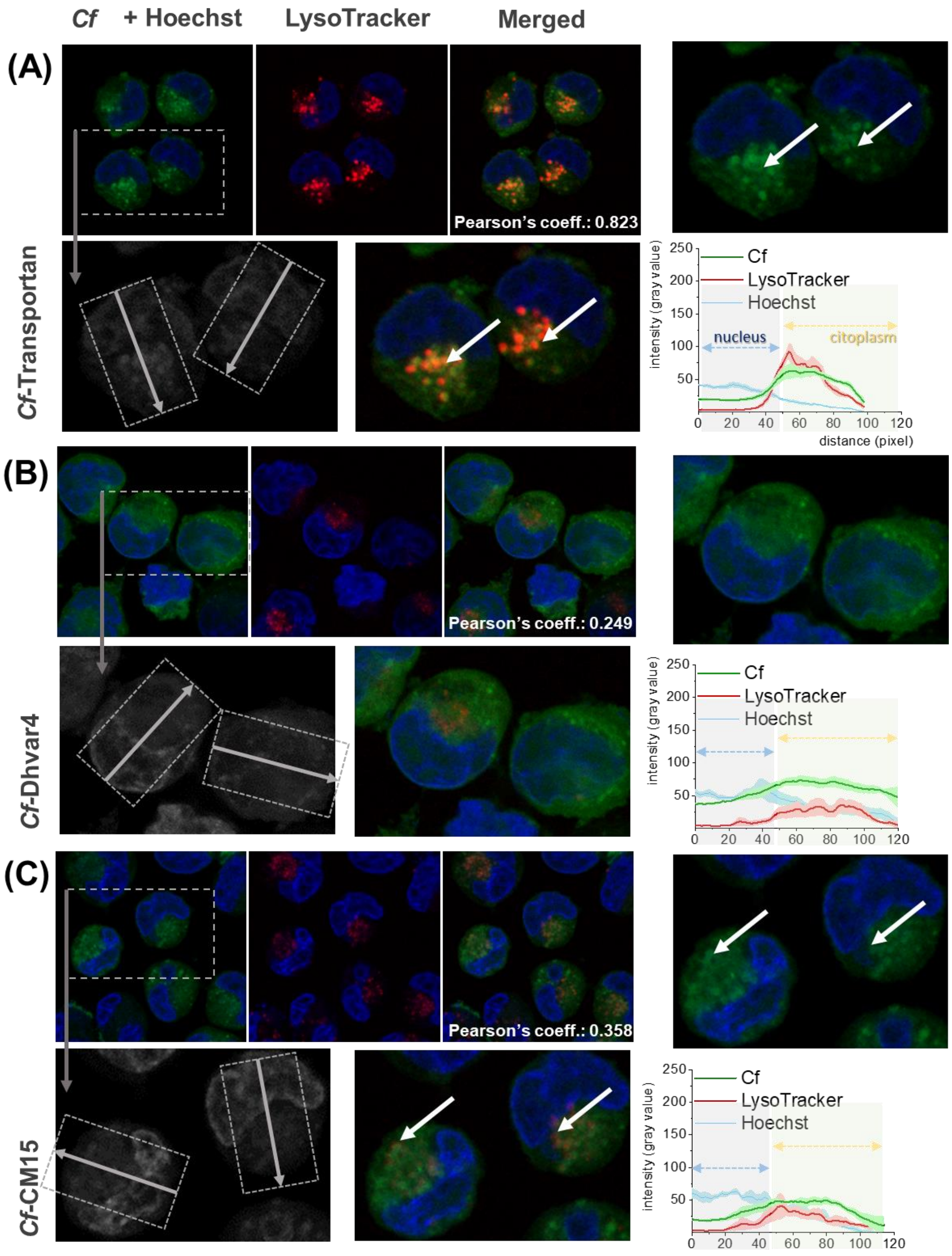

Supplementary Figure 3. *In vitro* intracellular localization of *Cf*-Transportan (panel **A)**; Cf-Dhvar4 (Panel **B**) and Cf-CM15 ((Panel **C**) visualized by CLSM. Cells were treated for 2 hrs with *Cf*-peptides (10 µM, green). Lysosomes were stained by LysoTracker Deep Red (red), nuclei were stained by Hoechst 33342 (blue). Imaging was performed by Zeiss LSM 710 system (upper part of each panels

and lower part enlargements). Laser intensity values were identical in case of all *Cf*-peptides. On the grayscale images the grey rectangles (covering the individual cells) were drawn with similar orientation (starting from the cells' nucleus towards their cytoplasm). The extracted gray values correspond to the intensity of a given pixel on a scale of 0 to 255. Line scan analysis was performed by NIH ImageJ software using the Plot Profile application with two representative cells/image (see enlargements in each panels). Standard error was calculated visualized by OriginPro 2018 software.

REFERENCES

1. Rogers, R.N. and S.K. Yasuda, *Rapid Microdetermination of Fluorine in Organic Compounds.* Anal Chem, 1959. **31**(4): p. 616-617.
2. Antonio, P.D., et al., *Scale-independent roughness value of cell membranes studied by means of AFM technique.* Biochimica Et Biophysica Acta-Biomembranes, 2012. **1818**(12): p. 3141-3148.
3. Dunn, K.W., M.M. Kamocka, and J.H. McDonald, *A practical guide to evaluating colocalization in biological microscopy.* American Journal of Physiology-Cell Physiology, 2011. **300**(4): p. C723-C742.
4. Bolte, S. and F.P. Cordelieres, *A guided tour into subcellular colocalization analysis in light microscopy.* Journal of Microscopy, 2006. **224**: p. 213-232.
